# Efficacy of *Ginkgo biloba* on parameters in glaucoma: A systematic review

**DOI:** 10.1371/journal.pone.0314644

**Published:** 2025-02-14

**Authors:** Julia Prinz, Verena Prokosch, Xiaosha Wang, Yuan Feng, Peter Walter, Matthias Fuest, Filippo Migliorini

**Affiliations:** 1 Department of Ophthalmology, University Hospital RWTH Aachen, Aachen, Germany; 2 Department of Ophthalmology, University Hospital of Cologne, Cologne, Germany; 3 Department of Orthopedics and Trauma Surgery, Academic Hospital of Bolzano (SABES-ASDAA), Bolzano, Italy; 4 Department of Life Sciences, Health, and Health Professions, Link Campus University, Rome, Italy; Brigham and Women’s Hospital, UNITED STATES OF AMERICA

## Abstract

**Purpose:**

This study aims to analyse the efficacy of GBE administration in patients with glaucoma and healthy volunteers.

**Methods:**

This systematic review was performed according to the PRISMA 2020 guidelines. All clinical studies investigating the efficacy of GBE administration on the intraocular pressure (IOP), the corrected pattern standard deviation (CPSD) and the mean deviation of visual field testing, and heart rate were considered. The weighted mean difference (MD) effect measure, 95% confidence interval (CI), and t-test were used for continuous variables.

**Results:**

Data from 8 studies, including 428 patients, were retrieved. The mean age of all patients was 51.1 ± 15.5 years. The median follow-up was 3.7 (IQR 9.4) months. The administration of GBE was not associated with an improvement in IOP (MD -1.5; 95%CI -7.1 to 9.6; P = 0.5), mean deviation (MD 0.7; 95%CI -9.4 to 8.2; P = 0.8), CPSD (MD -1.6; 95%CI -3.8 to 6.9; P = 0.5), or heart rate (MD -2.5; 95%CI -11.5 to 16.5; P = 0.4) from baseline to the last follow-up. There was no difference between GBE versus the control group in IOP (MD 1.1; 95%CI -5.7 to 3.5; P = 0.4), mean deviation (MD -0.4; 95%CI -9.1 to 9.9; P = 0.9), CPSD (MD 0.3; 95%CI -6.8 to 6.2; P = 0.9), and heart rate (MD -1.3; 95%CI -15.1 to 17.7; P = 0.8) at the last follow-up.

**Conclusion:**

Currently, the evidence is not sufficient to conclude that GBE affects IOP, mean deviation, CPSD, or heart rate in glaucoma patients and healthy volunteers. These conclusions must be interpreted with caution given the limitations of the reviewed studies, particularly the follow-up time of the included studies.

## Introduction

Glaucoma is a heterogeneous group of diseases characterised by apoptosis of retinal ganglion cells (RGCs) and their axons [1]. RGCs are the innermost neurons of the retina and transmit visual signals from the retina along axons that extend in the optic nerve to the brain [2]. Recently, it has been suggested that RGCs may enter a state of physiological dysfunction prior to apoptosis [2].

Elevated intraocular pressure (IOP) causes mechanical RGC and axonal damage and interrupts nutrient transmission [1]. Lowering IOP is the current mainstay of treatment but does not halt glaucoma progression in many patients [3]. Therefore, alternative treatment strategies independently of IOP to prevent or delay glaucomatous neurodegeneration are urgently warranted [4, 5]. Besides elevated IOP, advancing age [6], glutamate excitotoxicity [7], oxidative stress [8], endothelial [9] and mitochondrial [10] dysfunction, neurotrophin deficiency [11], and further risk factors have been identified to contribute to glaucoma progression. Recently, the role of vascular abnormalities in the pathogenesis of glaucoma has been highlighted, including reduced perfusion pressure, local vasospasms, and impaired retinal blood flow autoregulation [12–15].

In patients with dementia such as Alzheimer’s disease, standardised *Ginkgo biloba* extract (GBE) derived from dried leaves of the *Ginkgo* tree has been postulated to slow the progression of memory impairment [16]. GBE has been shown to play an important role in the treatment of several degenerative eye diseases, such as age-related macular degeneration, diabetic retinopathy, ischaemic retinal disease, and glaucoma [17, 18]. However, to date, the efficacy of GBE in these diseases is inconclusive [17, 19].

The rationale behind using GBE in these diseases is that GBE contains different flavonoids, terpene lactones, and organic acids [20]. Flavonoids are naturally occurring polyphenolic compounds that deliver electrons to free radicals and reduce the production of eicosanoids by inhibiting the activity of phospholipase A2 [21–23]. In addition to its antioxidative and anti-inflammatory properties, GBE has been shown to improve endothelium-dependent vasodilation, thereby increasing microcirculation and reversing vasospasms [23–25]. Also, GBE stabilises the inner mitochondrial membrane and increases the membrane potential, which restores the respiratory chain and increases the production of adenosine triphosphate [26, 27].

In previous studies, GBE has been shown to slow the progression of visual field damage and improve visual function in normal tension glaucoma (NTG) patients [28, 29]. However, to date, conclusive evidence of the efficacy of GBE on IOP, the mean deviation and the corrected pattern standard deviation (CPSD) of visual field testing, and the heart rate are lacking. This systematic review investigates first-time the impact of GBE on IOP, mean deviation, CPSD, and the heart rate in glaucoma patients and healthy volunteers.

## Material and methods

### Eligibility criteria

All the comparative studies evaluating the efficacy of oral GBE administration in patients with glaucoma and healthy volunteers were retrieved. Studies which compared GBE administration in isolation or combined with the standard therapy for glaucoma were considered. Studies combining GBE with other neuroprotective substances in the GBE group were excluded. Only studies that compared GBE administration with a control group of patients undergoing standard therapy, placebo or no therapy were considered. Given the authors’ language capabilities, English, Italian, German, Spanish, and French articles were eligible. Levels I to IV of evidence, according to the Oxford Centre of Evidence-Based Medicine [30], were considered. Reviews, opinions, letters, editorials, animal, in vitro, or biomechanics studies were not considered. A lack of quantitative data on the outcomes of interest led to an exclusion from the present study.

### Search strategy

This systematic review was conducted according to the 2020 PRISMA (Preferred Reporting Items for Systematic Reviews and Meta-Analyses) statement [31]. The PICO algorithm was preliminary established:

- P (Population): patients with glaucoma or healthy volunteers;
- I (Intervention): GBE supplementation (in addition to conventional therapy);
- C (Comparison): control group (standard therapy, placebo or no therapy);
- O (Outcomes): IOP, mean deviation, CPSD, heart rate.

In December 2023, the following databases were accessed: Google Scholar, Web of Science, PubMed, and Embase. No time constraints were used for the search. The following keywords were used in combination: *Ginkgo; Ginkgo biloba; Ginkgo biloba extract; glaucoma*, *open-angle glaucoma*, *OAG*, *primary open-angle glaucoma*, *POAG*, *secondary glaucoma*, *juvenile glaucoma*, *pseudoexfoliative glaucoma*, *normal tension glaucoma*, *NTG*, *angle closure glaucoma*, *pigmentary glaucoma*, *phacogenic glaucoma*, *neovascular glaucoma*, *intraocular pressure; visual field testing; mean deviation of visual field testing; corrected pattern standard deviation; ocular blood flow; optic nerve; neuroretinal rim; heart rate*.

### Selection and data collection

Two authors (JP; FM) independently performed the database search. All the resulting titles were screened, and the abstracts and full texts were accessed if suitable. Disagreements were discussed, and a third author made the final decision.

### Data items

Data extraction was conducted independently by two authors (JP, FM). The following data were extracted: study generalities (author, journal, year, design, level of evidence, and follow-up length) and patient demographics (number of patients, mean age, and women). If the studies reported data from several follow-up appointments, only data from the last follow-up were included for analysis. Data concerning the following parameters were retrieved: IOP, mean deviation, CPSD, and heart rate. The outcomes of interest were (1) to evaluate the effect of GBE on the IOP, mean deviation, CPSD, and heart rate and (2) to evaluate possible adverse events.

### Study risk of bias analysis

The risk of bias was assessed in accordance with the guidelines in the Cochrane Handbook for Systematic Reviews of Interventions [32]. Two authors (JP; FM) independently evaluated the risk of bias in the extracted studies. Randomised controlled trials (RCTs) were assessed using the risk of bias of the software Review Manager 5.3 (The Nordic Cochrane Collaboration, Copenhagen). The following endpoints were evaluated: detection, reporting, attrition, selection, performance, and other biases. The Risk of Bias in Nonrandomised Studies of Interventions (ROBINS-I) tool was used to assess the risk of bias in non-RCTs [33].

### Statistical analysis

Statistical analysis was conducted using the IBM SPSS software version 25. For descriptive statistics, the weighted mean, with the weights being the sample size, and standard deviation was used. The weighted mean and standard deviation were used to assess baseline comparability. Given the heterogeneity, data variability on follow-up was evaluated using the interquartile range (IQR). Between-group comparisons at the last follow-up were evaluated using the mean difference effect measure (MD) and unpaired t-test. Changes from baseline to the last follow-up were evaluated using the MD and unpaired t-test. The overall effect was considered statistically significant if P < 0.05.

## Results

### Literature search

**Fig 1:**
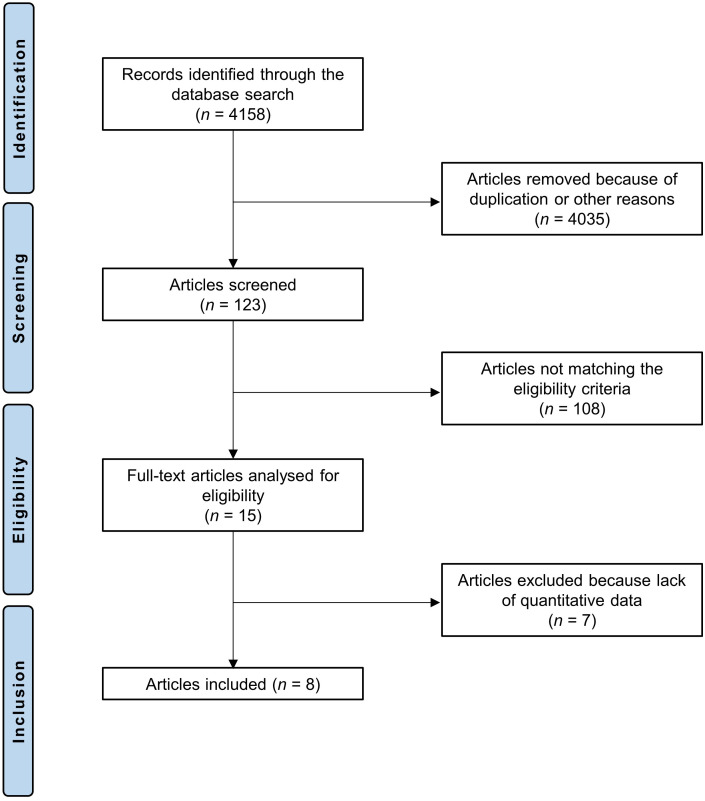
Flow chart of the literature search.

The literature search led to 4158 articles, of which 1102 were excluded due to duplication. A further 2933 studies were excluded based on the abstracts of the records. Thus, 123 full-text articles were screened, of which 108 studies were excluded because of the following reasons: type of study (N = 47), combination of different supplements (N = 8), language incompatibility (N = 4), or other reasons (N = 49). Seven studies did not report quantitative data under the endpoints of interest and were therefore not included in this study. Finally, eight studies were included. The literature search results are displayed in Fig 1 and S2 Table.

### Study risk of bias analysis

**Fig 2:**
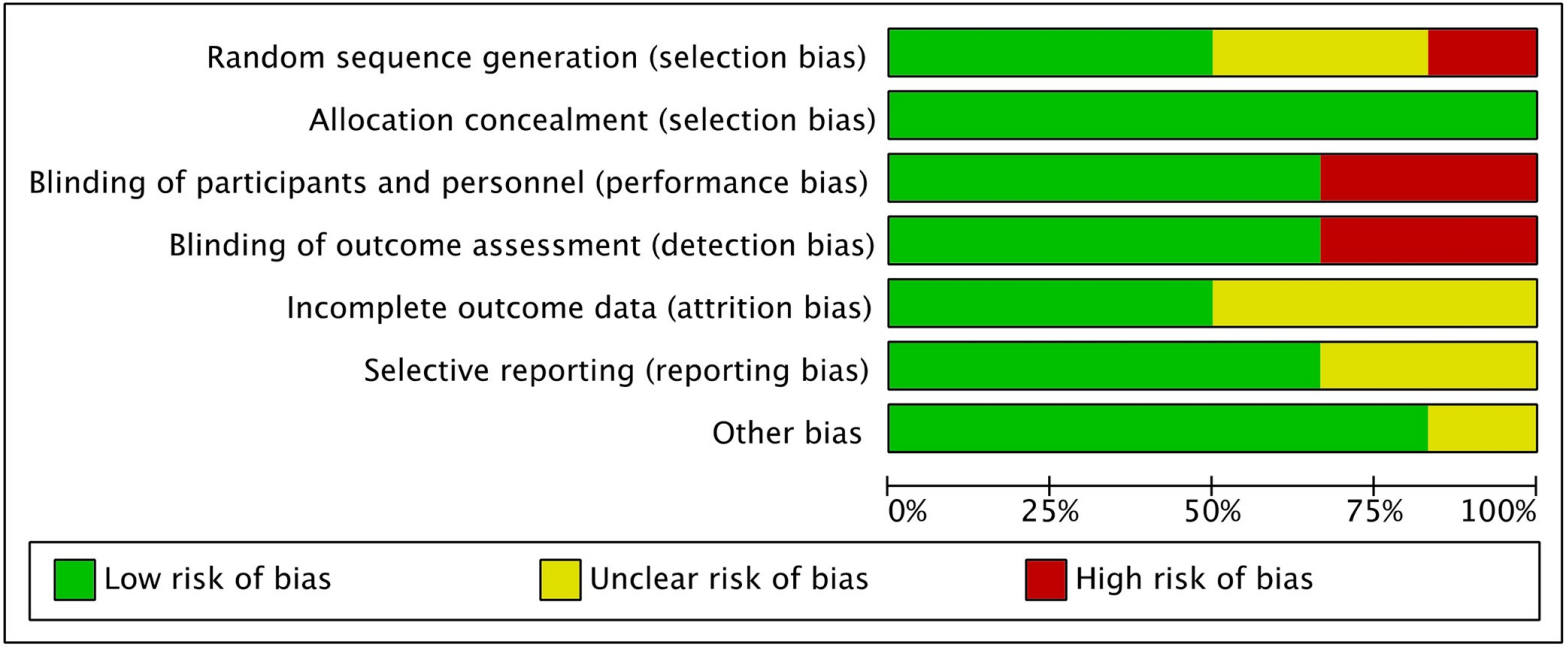
Methodological quality assessment.

The Cochrane risk of bias tool was conducted to evaluate the risk of bias of RCTs. Given the number of retrospective studies included in the present systematic review, the risk of selection bias was moderate. Few studies performed blinding, leading to a moderate risk of detection bias. The risk of attrition, reporting and other biases was moderate. In conclusion, the risk of bias graph evidenced a moderate quality of the methodological assessment of RCTs (Fig 2).

The ROBINS-I was performed to investigate the risk of bias of non-RCTs. The risks of bias due to confounding and deviation from the intended intervention were moderate. The risks of bias in the selection of participants for the study, bias in the classification of interventions and bias due to missing data were considered low to moderate, as was the risk of bias in the measurement of outcomes. Also, a low to moderate risk of bias was evidenced in the selection of the reported results. Overall, the quality of the methodological assessment was low to moderate (Table 1).

**Table 1:** Risk of Bias in Nonrandomised Studies of Interventions (ROBINS-I) evaluation.

Study	Bias due to confounding	Bias in selection of participants into the study	Bias in classification of interventions	Bias due to deviations from intended intervention	Bias due to missing data	Bias in measurement of outcomes	Bias in selection of the reported results	Overall risk of bias
Guo, 2014 [34]	*Low*	*Moderate*	*Low*	*Low*	*Low*	*Low*	*Low*	*Low*
Lee, 2013 [29]	*Moderate*	*Low*	*Moderate*	*Moderate*	*Moderate*	*Low*	*Moderate*	*Moderate*
Quaranta, 2003 [35]	*Low*	*Low*	*Moderate*	*Low*	*Low*	*Low*	*Low*	*Low*
Sabaner, 2021 [36]	*High*	*Moderate*	*Moderate*	*Moderate*	*Low*	*Moderate*	*Low*	*Moderate*
Sari, 2016 [37]	*Low*	*Low*	*Low*	*Moderate*	*Moderate*	*Low*	*Low*	*Moderate*
Shim, 2012 [28]	*High*	*Low*	*Moderate*	*High*	*Low*	*Low*	*Moderate*	*Moderate*

### Study characteristics

A total of 428 patients were included for analysis. 56% (241 of 428 patients) were women. The median follow-up was 3.7 (IQR 9.4) months. The mean age was 51.1 ± 15.5 years. Patients with NTG (n = 327), primary open-angle glaucoma (POAG, n = 30), and healthy volunteers (n = 71) were included. Demographic data of the patients are shown in Table 2 and S1 Table.

**Table 2:** Characteristics and patient baseline of the included studies (GBE: *Ginkgo biloba* extract, NTG: Normal tension glaucoma, POAG: Primary open-angle glaucoma, IOP: Intraocular pressure, mean deviation: Mean deviation of the visual field testing, CPSD: Corrected pattern standard deviation of visual field testing, NR: Not reported).

Author et al., year	Study design	Follow-up (*months*)	Group	Treatment	Patients (*n*)	Patient charact.	Mean age (years)	Women (*%*)	Endpoints
Chung, 1999 [38]	RCT	0.5	GBE	40 mg GBE, three times daily	5	Healthy volunteers	34.0	73	IOP, heart rate
			Placebo	placebo	6		34.0	73	
Guo, 2014 [34]	Retro-spective	3.7	GBE	40 mg GBE, three times daily	14	NTG	62.3	50	mean deviation, heart rate
			Placebo	placebo	14		65.1	36	
Lee, 2013 [29]	Retro-spective	147.6	GBE	80 mg GBE, twice daily	42	NTG	47.1	55	IOP
Park, 2011 [39]	RCT	0.9	GBE	80 mg GBE, twice daily	15	NTG	NR	53	IOP, CPSD
			Placebo	placebo	15		NR	73	
Quaranta, 2003 [35]	Retro-spective	2.7	GBE	40 mg GBE, three times daily	14	NTG	70.4	59	IOP, mean deviation, CPSD, heart rate
			Placebo	placebo	13		70.4	59	
Sabaner, 2021 [36]	Pro-spective	1.0	GBE	120 mg GBE daily	60	Healthy volunteers	20.6	53	IOP
Sari, 2016 [37]	Pro-spective	6.0	GBE	40 mg GBE, twice daily	20	POAG	54.6	70	IOP, mean deviation
			Placebo	placebo	10		54.9	60	
Shim, 2012 [28]	Retro-spective	23.8	GBE	80 mg GBE, twice daily	103	NTG	47.0	37	mean deviation
			Control	No treatment	97		52.3	37	

### Baseline comparability

At baseline, no significant differences were found in IOP (P = 0.5), mean deviation (P = 0.7), CPSD (P = 0.8), and heart rate (P = 0.8, Table 3) between the GBE and the control group.

**Table 3:** Baseline comparability between the *Ginkgo biloba extract* (GBE) and control groups. Data is presented as arithmetic mean and standard deviation. (IOP: intraocular pressure, mean deviation: mean deviation of the visual field testing, CPSD: corrected pattern standard deviation of visual field testing, bpm: beats per minute).

Endpoint	GBE	Control	P
IOP [mmHg]	15.3 ± 4.2	16.9 ± 5.1	0.5
Mean deviation [dB]	-7.3 ± 3.2	- 7.8 ± 4.1	0.7
CPSD	9.1 ± 2.6	8.4 ± 2.7	0.8
Heart rate [bpm]	70.3 ± 4.9	69.6 ± 5.2	0.8

### Result syntheses

The administration of GBE was not associated with an improvement in IOP (MD -1.5; 95%CI -7.1 to 9.6; P = 0.5), mean deviation (MD 0.7; 95%CI -9.4 to 8.2; P = 0.8), CPSD (MD -1.6; 95%CI -3.8 to 6.9; P = 0.5), or heart rate (MD -2.5; 95%CI -11.5 to 16.5; P = 0.4) from baseline to the last follow-up (Table 4).

**Table 4:** Evaluation of *Ginkgo biloba extract* (GBE) administration at baseline and the last follow-up (FU) in the GBE group. Data is presented as arithmetic mean and standard deviation (IOP: intraocular pressure, mean deviation: mean deviation of the visual field testing, CPSD: corrected pattern standard deviation of visual field testing, bpm: beats per minute, CI: confidence interval).

Endpoint	At baseline	Last FU	Mean difference	95%CI	P
IOP [mmHg]	15.3 ± 4.2	13.8 ± 1.2	-1.5	-7.1 to 9.6	0.5
Mean deviation [dB]	-7.3 ± 3.2	-6.6 ± 3.1	0.7	-9.4 to 8.2	0.8
CPSD	9.1 ± 2.6	7.5 ± 0.9	-1.6	-3.8 to 6.9	0.5
Heart rate [bpm]	70.3 ± 4.9	67.8 ± 5.2	-2.5	-11.5 to 16.5	0.4

At the last follow-up, there was no difference between GBE and the control group in IOP (MD 1.1; 95%CI -5.7 to 3.5; P = 0.4), mean deviation (MD -0.4; 95%CI -9.1 to 9.9; P = 0.9), CPSD (MD 0.3; 95%CI -6.8 to 6.2; P = 0.9), and heart rate (MD -1.3; 95%CI -15.1 to 17.7; P = 0.8). Table 5 shows these results in greater detail.

**Table 5:** Comparison of the *Ginkgo biloba extract* (GBE) and the control group. Data is presented as arithmetic mean and standard deviation. (IOP: intraocular pressure, mean deviation: mean deviation of the visual field testing, CPSD: corrected pattern standard deviation of visual field testing, bpm: beats per minute, CI: confidence interval).

Endpoint	GBE	Control	Mean difference	95%CI	*P*
IOP [mmHg]	13.8 ± 1.2	14.9 ± 2.0	1.1	-5.7 to 3.5	0.4
Mean deviation [dB]	-6.6 ± 3.1	-7.0 ± 3.7	-0.4	-9.1 to 9.9	0.9
CPSD	7.5 ± 0.9	7.8 ± 3.2	0.3	-6.8 to 6.2	0.9
Heart rate [bpm]	68.3 ± 6.1	67.0 ± 5.7	-1.3	-15.1 to 17.7	0.8

## Discussion

GBE administration did not significantly improve IOP, mean deviation, CPSD, or heart rate in patients with glaucoma and healthy volunteers after a mean follow-up of 23.3 (median 3.7) months.

The rationale behind investigating the efficacy of GBE on glaucoma parameters is its potential neuroprotective effect, which has been attributed to anti-oxidative, anti-inflammatory, anti-apoptotic, vaso-active, and angiogenetic properties [17, 40]. On a molecular level, GBE targets various signalling pathways that play a role in glaucoma, such as the protein-1 signalling pathway, which suppresses tumour necrosis factor α (TNF α) [41]. TNF α is a critical pro-inflammatory cytokine implicated in the apoptotic death of RGCs in glaucoma [42]. Also, GBE is involved in the activation of the adenosine monophosphate-activated protein kinase (AMPK) signalling pathway, affecting NFκB, mTOR, Nrf-2, and Wnt/β-catenin signalling pathways [40], which are implicated in the pathogenesis of glaucoma [43–45].

Vascular dysregulation and vasospasms have been postulated to contribute to the pathogenesis of glaucoma [46, 47]. To date, only a few studies have investigated the effect of GBE on ocular or peripapillary blood flow [36]. GBE has been shown to increase nitric oxide (NO) levels, which leads to vasodilation and increased blood flow via vasodilatory molecules, including histamine, bradykinin, substance P, and acetylcholine [17]. Also, GBE inhibits prostaglandin PGI2, leading to a decreased renin release and less vasoconstriction via the renin-angiotensin pathway [48]. In a study by Sabaner et al., a 4-week consumption of GBE led to an increase in peripapillary, superior, inferior, and temporal quadrant radial peripapillary capillary vascular density in healthy volunteers [36]. A prospective study found a significant increase in blood flow measured by a confocal scanning laser Doppler flowmeter in the GBE group compared to a placebo group, including 30 NTG patients [39]. Chung et al. found a significantly increased end-diastolic velocity in the ophthalmic artery measured by colour Doppler imaging in healthy volunteers following GBE treatment of 120 mg daily for two days. In contrast, no changes were witnessed in the placebo group [38]. In a study by Lee et al., the same dose of GBE administered for four weeks led to an increased microcirculatory blood velocity and flow [29]. Impaired vascular function significantly contributes to the progressive degeneration of RGCs and the optic nerve in glaucoma [15]. Emerging evidence suggests that GBE supplementation might be associated with increased ocular blood flow and reduced vasospasms, hypothetically slowing glaucoma progression. However, GBE was not associated with a significant improvement in mean deviation or CPSD in the present study, which might be attributable to the heterogeneous duration of GBE supplementation and follow-ups in the included studies as well as heterogeneous patient characteristics, including healthy volunteers, POAG, and NTG patients.

GBE administration resulted in a significant reduction of the heart rate in animal studies [49]. As fluctuations of IOP depending on the heart rate have been witnessed previously, the effects of neuroprotective agents on the heart rate might affect glaucoma progression [50]. A faster heart rate is associated with a lower neural tissue shear, which might limit axonal damage in the neuroretinal rim region [51, 52]. However, no significant effect of GBE on the heart rate was detected in the included studies [34, 35, 38].

None of the included studies evidenced significant changes using GBE on IOP in glaucoma patients or healthy volunteers [28, 29, 35–39].

Shim et al. [28] reported a significant improvement in visual acuity in NTG patients receiving 160 mg GBE daily for almost two years. Investigating healthy volunteers, Sabaner et al. found no changes in visual acuity following GBE treatment [36]. No further visual acuity outcomes were reported in the included studies. Given the lack of quantitative data for analysis, visual acuity could not be considered an endpoint of the present study.

Quaranta et al. investigated the effect of GBE on pre-existing visual field damage in patients with NTG. They found a significant improvement in mean deviation and CPSD after GBE treatment [35]. In a study by Lee et al. [29], GBE slowed the progression of visual field damage in NTG patients with a mean follow-up of 12.3 years. In previous studies by Park et al. and Guo et al., no significant impact of GBE therapy on the mean deviation was witnessed [34, 39]. Current evidence suggests that GBE supplementation may contribute to maintaining the visual field and that long-term treatment could even restore visual field defects in patients with glaucoma [29, 34, 39]. With a mean follow-up of 23.3 (median 3.7) months, visual field parameters were not worsening in patients receiving GBE in the included studies. However, there were no significant differences between the GBE and control group at the final follow-up, suggesting no superiority of GBE over placebo or no treatment in maintaining visual field defects. Guo et al. hypothesise that the treatment period in their study (4 weeks) was too short to demonstrate a clinical improvement in the visual field [34]. Additionally, the authors argue that the small sample size in their study may not have been sufficient to reveal the clinical effect of GBE on mean deviation [34]. Heterogeneity in patient characteristics, including ethnicity [34], type of glaucoma or healthy participants, and severity of glaucoma at the time of inclusion in the study, could also be responsible for different results between the individual studies. An important pitfall in the studies included is the duration and the frequency of visual field testing. It was long recognised that the observation period for trials of visual field preservation in patients with open-angle glaucoma is typically five years or longer to find visual field deterioration [53]. Importantly, Garway-Heath et al. recently demonstrated that a period of at least 24 months with repeated (clustered) visual field tests at the beginning and end of the observation period is required to detect visual field progression sufficiently [53, 54]. This duration or follow-up schedule was not used in the included studies. Therefore, the impact of GBE supplementation on visual field progression should be interpreted considering this important limitation.

The UK Glaucoma Treatment Study (UKGTS) was the first placebo-controlled RCT demonstrating the preservation of the visual field by a conventional IOP-lowering drug (latanoprost) in patients with open-angle glaucoma [53]. Visual field deterioration occurred in 15.2% of the patients treated with latanoprost and 25.6% without treatment (placebo group) [53]. However, in our study, there was no significant difference between patients receiving GBE combined with the standard therapy for glaucoma and the control group receiving the standard treatment only or in combination with placebo at the final follow-up.

The limited number of clinical studies included for analysis represents an important limitation of the present systematic review. Another important limitation is the retrospective design of half of the included studies. Given the limited data available in the literature, the analyses were conducted irrespective of the GBE dose or treatment duration. This study includes NTG, POAG patients, and healthy volunteers. Given the lack of quantitative data, no subgroups were possible to investigate. In addition, the heterogeneous length of follow-up might also limit the reliability of our results. Another important limitation is the short follow-up time for the included studies. Hypothetically, only long-term therapy, possibly over several years, with GBE has a positive effect on the visual field and glaucoma progression. Given the limited data available, a stratified analysis to determine the minimum required duration of therapy with GBE was not possible. Future studies should investigate the minimum treatment necessary and follow-up durations further. Furthermore, based on the different investigators and implementation options, it cannot be ruled out that the parameters investigated in this study, especially visual field testing, were not evaluated identically in all included studies. Given the limited data available for inclusion and the poor evidence of the included studies, a formal meta-analysis was not conducted. Additional comparative studies are required. Given the paucity of data available, a formal meta-analysis was not conducted. Moreover, variability in the content of the placebo and the unpredictable nature intrinsic to placebo administration might exert an influence on the reliability of the results. Therefore, additional research using an active comparator is necessary to establish the potential of GBE on glaucoma parameters including visual field damage progression. The studies included in this systematic review investigated the efficacy of GBE as an adjuvant therapy only in NTG, POAG, or healthy participants. Therefore, our results cannot be generalised to all glaucoma subtypes. For a better understanding of the impact of GBE as a preventive or adjunctive neuroprotective therapy in healthy patients or patients with glaucoma, future larger cohort studies should focus on long-term outcomes. Moreover, further parameters which indicate glaucoma progression, such as the peripapillary retinal nerve fibre layer [55] or Bruch’s membrane opening-minimum rim width [56], should be considered.

## Conclusion

GBE did not significantly improve IOP, the mean deviation and CPSD of visual field testing, or heart rate in patients with NTG, POAG, or healthy volunteers. These conclusions must be interpreted within the limitations of the present study, in particular, the overall too-short follow-up time of the included studies to detect visual field changes validly. Future large cohort randomised controlled trials are warranted to further establish the role of GBE in the different glaucoma subtypes.

## Supporting information

S1 Checklist (DOCX)

S1 Table Generalities of the included studies and values included in the analyses (GBE: *Ginkgo biloba* extract, NTG: Normal tension glaucoma, POAG: Primary open-angle glaucoma, IOP: Intraocular pressure, pre: Pre-treatment, post: Post-treatment, MD: Mean deviation of the visual field testing, CPSD: Corrected pattern standard deviation of visual field testing, bpm: Beats per minute, NR: Not reported). All data was extracted in December 2023.
(DOCX)

S2 Table Results of the literature search. (PDF)
