# Supplementary material for: Efficacy of Ginkgo biloba on parameters in glaucoma: A systematic review
Source: PLoS One. 2025 Feb 14;20(2):e0314644. doi: 10.1371/journal.pone.0314644 (PMC11828365; doi:10.1371/journal.pone.0314644)
Supplement: S1 Table — All data was extracted in December 2023. (DOCX) [file pone.0314644.s002.docx]

| **Author et al., year** | **Study design** | **Follow-up (*months*)** | **Group** | **Treatment** | **Patients (*n*)** | **Patient characteristics** | **Mean age**  **(years)** | **Women (*%*)** | **End-points** | **IOP pre (mmHg)** | **IOP post (mmHg)** | **MD pre (dB)** | **MD post**  **(dB)** | **CPSD pre** | **CPSD post** | **Heart rate pre (bpm)** | **Heart rate post (bpm)** |
| --- | --- | --- | --- | --- | --- | --- | --- | --- | --- | --- | --- | --- | --- | --- | --- | --- | --- |
| Chung, 1999 (33) | RCT | 0.5 | GBE | 40 mg GBE, three times daily, two days | 5 | Healthy volunteers | 34.0 | 73 | IOP, heart rate | 13.5 ± 0.9 | 14.0 ± 1.0 |  |  | 7.24 | 6.79 | 74 ± 2 | 70 ± 2 |
|  |  |  | Placebo | placebo | 6 |  | 34.0 | 73 |  | 14.6 ± 0.8 | 14.0 ± 1.0 |  |  | 6.46 | 5.58 | 75 ± 2 | 71 ± 4 |
| Guo, 2014 (29) | Retro-spective | 3.7 | GBE | 40 mg GBE, three times daily | 14 | NTG | 62.3 | 50 | MD, heart rate |  |  | -8.16 ± 7.22 | -8.20 ± 6.82 |  |  | 74.5 ± 10.6 | 73.4 ± 10.4 |
|  |  |  | Placebo | placebo | 14 |  | 65.1 | 36 |  |  |  | -5.27 ± 4.28 | -5.0 ± 4.28 |  |  | 69.1 ± 8.6 |  |
| Lee, 2013 (24) | Retro-spective | 147.6 | GBE | 80 mg GBE, twice daily | 42 | NTG | 47.1 | 55 | IOP | 14.4 ± 1.6 | 14.3 ± 1.3 |  |  |  |  |  |  |
| Park, 2011 (34) | RCT | 0.9 | GBE | 80 mg GBE, twice daily | 15 | NTG | NR | 53 | IOP, CPSD | 12.6 | 12.4 |  |  |  |  |  |  |
|  |  |  | Placebo | placebo | 15 |  | NR | 73 |  | 13.1 | 13.0 |  |  |  |  |  |  |
| Quaranta, 2003 (30) | Retro-spective | 2.7 | GBE | 40 mg GBE, three times daily, four weeks | 14 | NTG | 70.4 | 59 | IOP, MD, CPSD, heart rate | 14.2 ± 1.2 |  | -11.4 ± 1.6 | -8.8 ± 1.3 | 10.93 ± 1.06 | 8.13 ± 1.06 | 64.6 ± 3.8 | 61.6 ± 3.4 |
|  |  |  | Placebo | placebo | 13 |  | 70.4 | 59 |  | 15.1 ± 2.6 | 14.8 ± 3.3 | -11.0 ± 1.2 | -11.1 ± 1.1 | 10.33 ± 1.04 | 10.05 ± 1.06 | 64.6 ± 3.8 | 63.0 ± 4.1 |
| Sabaner, 2021 (31) | Pro-spective | 1.0 | GBE | 120 mg GBE daily, four weeks | 60 | Healthy volunteers | 20.6 | 53 | IOP | 12.96 ± 2.36 | 12.76 ± 2.01 |  |  |  |  |  |  |
| Sari, 2016 (32) | Pro-spective | 6.0 | GBE | 40 mg GBE, twice daily | 20 | POAG | 54.6 | 70 | IOP, MD | 25.3 ± 2.92 | 15.48 ± 1.9 | -3.25 ± 0.02 | -2.39 ± 0.07 |  |  |  |  |
|  |  |  | Placebo | placebo | 10 |  | 54.9 | 60 |  | 25.95 ± 3.13 | 17.69 ± 1.32 | -3.32 ± 0.06 | -2.91 ± 0.02 |  |  |  |  |
| Shim, 2012 (23) | Retro-spective | 23.8 | GBE | 80 mg GBE, twice daily | 103 | NTG | 47.0 | 37 | MD |  |  | -5.25 ± 6.13 | -4.31 ± 5.60 |  |  |  |  |
|  |  |  | Control | No treatment | 97 |  | 52.3 | 37 |  |  |  | -5.41 ± 4.64 | -5.06 ± 6.32 |  |  |  |  |

**Supplemental Table 1:** Generalities of the included studies and values included in the analyses (GBE: *Ginkgo biloba* extract, NTG: normal tension glaucoma, POAG: primary open-angle glaucoma, IOP: intraocular pressure, pre: pre-treatment, post: post-treatment, MD: mean deviation of the visual field testing, CPSD: corrected pattern standard deviation of visual field testing, bpm: beats per minute, NR: not reported). All data was extracted in December 2023.
