# Supplementary material for: Efficacy of Ginkgo biloba on parameters in glaucoma: A systematic review
Source: PLoS One. 2025 Feb 14;20(2):e0314644. doi: 10.1371/journal.pone.0314644 (PMC11828365; doi:10.1371/journal.pone.0314644)
Supplement: S2 Table — (PDF) [file pone.0314644.s003.pdf]

Supplemental Table 2: Results of the literature search.

Author (et al)	Year		Journal	Title	Inclusion?	Reason
Schumer	1994	1	Archives of Ophthalmology	The nerve of glaucoma!	no	other / not on topic
Pritz-Hohmeier	1994	2	Ophthalmic Research	Effect of in vivo Application of the Ginkgo biloba Extract EGb 761 (Rökan®) on the Susceptibility of Mammalian Retinal Cells to Proteolytic Enzymes	no	study design / no quantitative data under the endpoints of interest
Zink	1998	3	American family physician	Herbal 'health'products: what family physicians need to know	no	not on topic
Greener	1998	4	Inpharma weekly	Sights set on neuroprotection for glaucoma	no	study design / no quantitative data under the endpoints of interest
Greenfield	1999	5	American journal of ophthalmology	Hyphema associated with pupillary dilation in a patient with exfoliation glaucoma and warfarin therapy	no	other / not on topic
Osborne	1999	6	Survey of ophthalmology	Neuroprotection in relation to retinal ischemia and relevance to glaucoma	no	other / not on topic
Spear	1999	7	Review of Optometry	Controversies in glaucoma care	no	study design / no quantitative data under the endpoints of interest
Trad	1999	8	Review of Optometry	Is Neuroprotection the Next Wave In Glaucoma Management?	no	study design / no quantitative data under the endpoints of interest
Cupp	1999	9	American familiy physician	Herbal remedies: adverse effects and drug interactions	no	study design / no quantitative data under the endpoints of interest
Chung	1999	10	Journal of Ocular Pharmacology an	Ginkgo biloba Extract Increases Ocular Blood Flow Velocity	yes	
Ritch	2000	11	Medical hypotheses	Potential role for Ginkgo biloba extract in the treatment of glaucoma	no	no quantitative data under the endpoints of interest
Williams	2000	12	Review of optometry	What's New in Glaucoma	no	other / not on topic
Kudolo	2000	13	The Journal of Clinical Pharmacy	The Effect of 3-Month Ingestion of Ginkgo biloba Extract on Pancreatic β-Cell Function in Response to Glucose Loading in Normal Glucose Tolerant	no	other / not on topic
Ritch	2000	14	Current opinion in ophthalmology	Neuroprotection: is it already applicable to glaucoma therapy?	no	study design / no quantitative data under the endpoints of interest
Juarez	2000	15	European Journal of Ophthalmolog	Experimental retinopathy of prematurity: angiostatic inhibition by nimodipine, ginkgo-biloba, and dipyridamole, and response to different growth fa	no	study design / no quantitative data under the endpoints of interest
Quinn	2000	16	Review of Optometry	Glaucoma without pressure	no	study design / no quantitative data under the endpoints of interest
Rhee	2001	17	Survey of Ophthalmology	Complementary and Alternative Medicine for Glaucoma	no	Different supplements
Head	2001	18	Alternative Medicine Review	Natural therapies for ocular disorders part two: cataracts and glaucoma	no	study design / no quantitative data under the endpoints of interest
Granger	2001	19	Age and ageing	Ginkgo biloba precipitating epileptic seizures	no	study design / no quantitative data under the endpoints of interest
Harris	2001	20	Current opinion in ophthalmology	The impact of glaucoma medication on parameters of ocular perfusion	no	study design / no quantitative data under the endpoints of interest
McKenna	2001	21	Alternative Therapies in Health and	Efficacy, safety, and use of Ginkgo biloba in clinical and preclinical applications	no	study design / no quantitative data under the endpoints of interest
Vessani	2001	22	IOVS	Effect of ginkgo biloba on the outcome of visual fields of patients with primary open-angle glaucoma	no	study design / no quantitative data under the endpoints of interest
Elovic	2001	23	The Journal of Head Trauma	Ginkgo biloba: applications in traumatic brain injury	no	study design / no quantitative data under the endpoints of interest
Gurses-Ozden	2002	24	IOVS	Ginkgo Biloba Extract Does Not Alter Peripapillary Retinal Hemodynamics Using Heidelberg Retina Flowmetry In Open Angle Glaucoma	no	no quantitative data under the endpoints of interest
Meletis	2002	25	Alternative & Complementary Thera	Alternative and Complementary Approaches to Treating Common Ocular Disorders: Part 1-Cataracts and Glaucoma	no	not on topic
Savickiene	2002	26	Medicina (Kaunas)	[Importance of biologically active components and plants in the prevention of complications of diabetes mellitus].	no	other (study design)
Thiagarajan	2002	27	Experimental Eye Research	Molecular and cellular assessment of ginkgo biloba extract as a possible ophthalmic drug	no	study design / no quantitative data under the endpoints of interest
Drago	2002	28	Journal of Ocular Pharmacology an	Pharmacokinetics and bioavailability of a Ginkgo biloba extract	no	study design / no quantitative data under the endpoints of interest
Meletis	2002	29	Alternative & Complementary Thera	Alternative and Complementary Approaches to Treating Common Ocular Disorders: Part 1-Cataracts and Glaucoma	no	study design / no quantitative data under the endpoints of interest
Rhee	2002	30	Ophthalmology	Prevalence of the use of complementary and alternative medicine for glaucoma	no	study design / no quantitative data under the endpoints of interest
Costa	2003	31	Progress in Retinal and Eye Researc	The effects of antiglaucoma and systemic medications on ocular blood flow	no	not on topic
Ness	2003	32	The Journals of Gerontology: Series	“Polyherbacy”: herbal supplements as a form of polypharmacy in older adults	no	not on topic
Guttman	2003	33	Ophthalmology Times	Therapeutic decisions for glaucoma patients weigh benefits: specialists cover latest medical, laser therapy options for managing this chronic disea	no	study design / no quantitative data under the endpoints of interest
Quaranta	2003	34	Ophthalmology	Effect of Ginkgo biloba extract on preexisting visual field damage in normal tension glaucoma	yes	
Lai	2003	35	HKU Theses Online (HKUTO)	Neuroprotective effect of Ginkgo biloba extract on retinal ganglion cells in a rat glaucoma model	no	study design / no quantitative data under the endpoints of interest
Han	2003	36	Journal of the Korean Ophthalmolo	The effects of oral Ginkgo biloba extract on visual field change in patients with NTG	no	study design / no quantitative data under the endpoints of interest
Kaushik	2003	37	Journal of postgraduate medicine	Neuroprotection in glaucoma	no	study design / no quantitative data under the endpoints of interest
Marcic	2003	38	Current opinion in ophthalmology	Neuroprotection in glaucoma: a model for neuroprotection in optic neuropathies	no	study design / no quantitative data under the endpoints of interest
Carenini	2004	39	IOVS	Effects of Ginkgo biloba extract (GBE) on ocular blood flow in primary open angle glaucoma patients	no	no quantitative data under the endpoints of interest
Hirooka	2004	40	Current eye research	The Ginkgo biloba extract (EGb 761) provides a neuroprotective effect on retinal ganglion cells in a rat model of chronic glaucoma	no	study design / no quantitative data under the endpoints of interest
Oliff	2004	41	HerbalGram	Ginkgo Extract Reduces Visual Field Damage in Patients with Glaucoma.	no	study design / no quantitative data under the endpoints of interest
Fraunfelder	2004	42	American Journal of Ophthalmolog	Ocular side effects from herbal medicines and nutritional supplements	no	study design / no quantitative data under the endpoints of interest
Bone	2004	43	Townsend Letter for Doctors and Pa	Ginkgo and glaucoma	no	study design / no quantitative data under the endpoints of interest
Dubey	2004	44	Kathmandu University Medical Jour	Ginkgo biloba--an appraisal.	no	study design / no quantitative data under the endpoints of interest
Thienprasiddhi	2004	45	IOVS	Effect of Ginkgo Biloba on the Multifocal Visual Evoked Potential Responses in Patients with Normal Tension Glaucoma	no	study design / no quantitative data under the endpoints of interest
Ilieva	2004	46	Experimental Eye Research	The effects of Ginkgo biloba extract on lipopolysaccharide-induced inflammation in vitro and in vivo	no	study design / no quantitative data under the endpoints of interest
Wang	2005	47	Chinese medical journal	Protective effects of Ginkgo biloba extract 761 against glutamate-induced neurotoxicity in cultured retinal neuron	no	study design / no quantitative data under the endpoints of interest
Ritch	2005	48	Ophthalmology Clinics of North Am	Complementary therapy for the treatment of glaucoma: a perspective.	no	study design / no quantitative data under the endpoints of interest
Kuehn	2005	49	development	Retinal ganglion cell death in glaucoma: mechanisms and neuroprotective strategies.	no	study design / no quantitative data under the endpoints of interest
Omoti	2005	50	Nigerian journal of clinical practice	A review of the choice of therapy in primary open angle glaucoma	no	study design / no quantitative data under the endpoints of interest
Orgül	2005	51	Ophthalmologica	Therapeutic strategies for normal-tension glaucoma	no	study design / no quantitative data under the endpoints of interest
Page	2005	52	The Journals of Gerontology	Electrophysiological analysis of the effects of ginkgo biloba on visual processing in older healthy adults	no	study design / no quantitative data under the endpoints of interest
Park	2005	53	Journal of the Korean Medical Asso	Medical treatment of glaucoma	no	study design / no quantitative data under the endpoints of interest
Luksch	2005	54	British journal of ophthalmology	Effect of nimodipine on ocular blood flow and colour contrast sensitivity in patients with normal tension glaucoma	no	study design / no quantitative data under the endpoints of interest
West	2006	55	American journal of ophthalmology	Evidence for the use of nutritional supplements and herbal medicines in common eye diseases	no	no quantitative data under the endpoints of interest
Mazza	2006	56	European Journal of Neurology	Ginkgo biloba and donepezil: a comparison in the treatment of Alzheimer's dementia in a randomized placebo-controlled double-blind study	no	not on topic
Zagaria	2006	57	Journal of Modern Pharmacy	OTC medication use in the elderly	no	not on topic

Izzotti	2006	58	Recenti Prog Med	[Open angle glaucoma: epidemiology, pathogenesis and prevention].	no	other (study design)
Chan	2006	59	Eye	Delayed retrobulbar haemorrhage after Ahmed glaucoma implant: a case report	no	other / not on topic
Pache	2006	60	Survey of ophthalmology	A sick eye in a sick body? Systemic findings in patients with primary open-angle glaucoma	no	other / not on topic
Kumarasamy	2006	61	European Journal of Inflammation	Glaucoma: Current and Developing Concepts for Inflammation, Pathogenesis and Treatment	no	study design / no quantitative data under the endpoints of interest
Izzotti	2006	62	Mutation Research	The role of oxidative stress in glaucoma	no	study design / no quantitative data under the endpoints of interest
Wolf	2006	63	Drugs in R & D	Does Ginkgo biloba Special Extract EGb 761® Provide Additional Effects on Coagulation and Bleeding when Added to Acetylsalicylic Acid 500mg Da	no	study design / no quantitative data under the endpoints of interest
Sadler	2006	64	Canadian pharmacist Journal	Gingko Biloba: Practical Management of Adverse Effects and Drug Interactions	no	study design / no quantitative data under the endpoints of interest
Dumitrica	2007	65	Oftalmologica	[Ginkgo biloba in glaucoma]	no	Language incompatibility
Wimpissinger	2007	66	Acta Ophthalmologica Scandinavic	Influence of Ginkgo biloba on ocular blood flow	no	no quantitative data under the endpoints of interest
Cobb	2007	67	Eye	The effect of aspirin and warfarin therapy in trabeculectomy	no	not on topic
Leung	2007	68	Medical Bulletin	Normal Tension Glaucoma-a Sick Eye in a Sick Body	no	other / not on topic
Dorairaj	2007	69	Explore	Visual improvement in a patient taking ginkgo biloba extract: a case study	no	study design / no quantitative data under the endpoints of interest
Mozaffarieh	2007	70	Survey of Ophthalmology	Is there more to glaucoma treatment than lowering IOP?	no	study design / no quantitative data under the endpoints of interest
Melinte	2007	71	Journal Français d'Ophtalmologie	563 Ginkgo biloba et le glaucome	no	study design / no quantitative data under the endpoints of interest
Mozaffarieh	2007	72	Expert Opinion on Emerging Drugs	A novel perspective on natural therapeutic approaches in glaucoma therapy	no	study design / no quantitative data under the endpoints of interest
Childlow	2007	73	Drugs	Pharmacological neuroprotection for glaucoma	no	study design / no quantitative data under the endpoints of interest
Dumitrică	2007	74	Oftalmologia	Ginkgo biloba in glaucoma	no	study design / no quantitative data under the endpoints of interest
Sekretar	2007	75	Journal of food and nutrition resear	Determination of the antioxidant activity of Ginkgo biloba leaves extract	no	study design / no quantitative data under the endpoints of interest
Orgül	2007	76	British journal of ophthalmology	Blood flow in glaucoma	no	study design / no quantitative data under the endpoints of interest
Vulsteke	2008	77	Graefe's Archive for Clinical and Ex	Correlation between ocular pulse amplitude measured by dynamic contour tonometer and visual field defects	no	no quantitative data under the endpoints of interest
Reitsamer	2008	78	Spektrum der Augenheilkunde	Therapy of blood flow impairments in glaucoma patients between theory and practical approaches?	no	not on topic
Tsai	2008	79	Canadian journal of ophthalmology	Influencing ocular blood flow in glaucoma patients: the cardiovascular system and healthy lifestyle choices	no	other / not on topic
Tataru	2008	80	Revista Medico-chirurgicala a Soci	Glaucoma--neurodegenerative disease	no	other / not on topic
Jia	2008	81	Laboratory Sciences	Effect of topical Ginkgo biloba extract on steroid-induced changes in the trabecular meshwork and intraocular pressure	no	study design / no quantitative data under the endpoints of interest
Mozaffarieh	2008	82	Survey of Ophthalmology	The potential value of natural antioxidative treatment in glaucoma	no	study design / no quantitative data under the endpoints of interest
Gunasekera	2008	83	Ophthalmology	Systematic internet-based review of complementary and alternative medicine for glaucoma	no	study design / no quantitative data under the endpoints of interest
Wu	2008	84	Genitourinary, Renal and Endocrine	Ginkgo biloba extract prevents against apoptosis induced by high glucose in human lens epithelial cells	no	study design / no quantitative data under the endpoints of interest
Zou	2008	85	International journal of ophthalmol	New ideas for medical therapy of glaucoma in the future	no	study design / no quantitative data under the endpoints of interest
Gupta	2008	86	Indian journal of ophthalmology	Recent advances in pharmacotherapy of glaucoma	no	study design / no quantitative data under the endpoints of interest
Cheung	2008	87	Optometry and Vision Science	Neuroprotection in glaucoma: drug-based approaches	no	study design / no quantitative data under the endpoints of interest
Sacca	2008	88	Progress in Brain Research	Oxidative stress and glaucoma: injury in the anterior segment of the eye	no	study design / no quantitative data under the endpoints of interest
Rhone	2008	89	Nutrition reviews	Phytochemicals and age-related eye diseases	no	study design / no quantitative data under the endpoints of interest
Saleem	2008	90	Stroke	Ginkgo Biloba Extract Neuroprotective Action Is Dependent on Heme Oxygenase 1 in Ischemic Reperfusion Brain Injury	no	study design / no quantitative data under the endpoints of interest
Khoury	2009	91	J Glaucoma	The Association Between Self-reported Glaucoma and Ginkgo Biloba Use	no	no quantitative data under the endpoints of interest
Khoury	2009	92	J Glaucoma	The Association Between Self-reported Glaucoma and Ginkgo Biloba Use	no	study design / no quantitative data under the endpoints of interest
Ma	2009	93	Eye	Dosage dependence of the effect of Ginkgo biloba on the rat retinal ganglion cell survival after optic nerve crush	no	study design / no quantitative data under the endpoints of interest
King	2009	94	British Journal of Ophthalmology	Should we be considering selenium in glaucoma?	no	study design / no quantitative data under the endpoints of interest
Pinal	2009	95	Eye Research Developments	Current trends in glaucoma: what about neuroprotection?	no	study design / no quantitative data under the endpoints of interest
Leung	2009	96	Hong Kong Journal of Ophthalmolo	Tips for management of normal tension glaucoma	no	study design / no quantitative data under the endpoints of interest
Vyas	2009	97	Review of Optometry	Nutritional therapeutics for the eye: nutritional supplements are playing a more prominent role in the treatment of AMD, cataract and glaucoma	no	study design / no quantitative data under the endpoints of interest
Neiberg	2009	98	Eye Care Review	Neuroprotection and Glaucoma.	no	study design / no quantitative data under the endpoints of interest
Perez	2009	99	Puerto Rico Health Sciences Journa	Can ginkgo biloba combat diseases?	no	study design / no quantitative data under the endpoints of interest
Ou	2009	100	Journal of applied physiology	Ginkgo biloba extract attenuates oxLDL-induced oxidative functional damages in endothelial cells	no	study design / no quantitative data under the endpoints of interest
Mozaffarieh	2010	101	EPMA Journal	Targeted preventive measures and advanced approaches in personalised treatment of glaucoma neuropathy	no	other / not on topic
Eke	2010	102	Current Anaesthesia & Critical Care	Anaesthesia for glaucoma surgery	no	other / not on topic
Yuki	2010	103	Graefe's Archive for Clinical and Ex	Reduced-serum vitamin C and increased uric acid levels in normal-tension glaucoma	no	other / not on topic
Zhang	2010	104	Glaucoma Medical Therapy	Nonprescribed Systemic Drugs and Therapies	no	study design / no quantitative data under the endpoints of interest
Aihara	2010	105	FitEyes	Non-pharmaceutical medications and approaches to glaucoma (all articles)	no	study design / no quantitative data under the endpoints of interest
Ma	2010	106	Acta ophthalmologica	The effect of ginkgo biloba on the rat retinal ganglion cell survival in the optic nerve crush model	no	study design / no quantitative data under the endpoints of interest
Zelefsky	2010	107	The Glaucoma Book	Alternative and non-traditional treatments of glaucoma	no	study design / no quantitative data under the endpoints of interest
Baltmr	2010	108	IOVS	Potential Targets for Ginkgo Biloba Neuroprotective Effects in a Rat Glaucoma Model; Cytochrome c, APP and Aβeta	no	study design / no quantitative data under the endpoints of interest
Jose Abad	2010	109	ingenta	An Update on Drug Interactions with the Herbal Medicine Ginkgo biloba	no	study design / no quantitative data under the endpoints of interest
Wierzbowska	2010	110	Med Sci Monit	Future possibilities in glaucoma therapy	no	study design / no quantitative data under the endpoints of interest
Engin	2010	111	Townsend Letter for Doctors and Pa	Variability of serum oxidative stress biomarkers relative to biochemical data and clinical parameters of glaucoma patients	no	study design / no quantitative data under the endpoints of interest
Chua	2010	112	Expert Review of Ophthalmology	Neuroprotective agents in glaucoma therapy: Recent developments and future directions	no	study design / no quantitative data under the endpoints of interest
Velpandian	2010	113	Drugs in R & D	Closed Gateways—Can Neuroprotectants Shield the Retina in Glaucoma?	no	study design / no quantitative data under the endpoints of interest
Engin	2010	114	Acta Ophthalmologica	Vasoregulatory and neuroprotective effects of oral antioxidants against glaucomateus damage	no	study design / no quantitative data under the endpoints of interest
Kim	2010	115	Journal of Ginseng Research	Effect of Korean red ginseng supplementation on ocular blood flow in patients with glaucoma	no	study design / no quantitative data under the endpoints of interest
Rosenstein	2010	116	Journal of Pineal Research	Melatonin as a therapeutic tool in ophthalmology: implications for glaucoma and uveitis	no	study design / no quantitative data under the endpoints of interest
Dahlmann-Noor	2010	117	Drug discovery today	Strategies for optic nerve rescue and regeneration in glaucoma and other optic neuropathies	no	study design / no quantitative data under the endpoints of interest

Stefan	2011	118	Oftalmologica	[Extract of Ginkgo biloba in glaucoma].	no	Language incompatibility
Field	2011	119	Physiology & behavior	Consumption of cocoa flavanols results in an acute improvement in visual and cognitive functions	no	not on topic
Parikh	2011	120	Indian Journal of Ophthalmology	Alternative therapy in glaucoma management: is there any role?	no	study design / no quantitative data under the endpoints of interest
Pinto	2011	121	Glaucoma-Basic and Clinical Conc	A Vascular Approach to Glaucoma	no	study design / no quantitative data under the endpoints of interest
Wilkinson	2011	122	Drugs	Use of herbal medicines and nutritional supplements in ocular disorders: an evidence-based review	no	study design / no quantitative data under the endpoints of interest
Wang	2011	123	[Zhonghua yan ke za Zhi] Chinese J	The protective effects of ginkgo biloba extract on cultured human retinal ganglion cells	no	study design / no quantitative data under the endpoints of interest
Vasudevan	2011	124	Indian journal of ophthalmology	Neuroprotection in glaucoma	no	study design / no quantitative data under the endpoints of interest
Awoyesuku	2011	125	The Nigerian Health Journal	Neuroprotection in glaucoma: a review	no	study design / no quantitative data under the endpoints of interest
Samples	2011	126	Prospects	Prospects for neuroprotection in glaucoma	no	study design / no quantitative data under the endpoints of interest
Kumar	2011	127	J. Res. Educ. Indian Med	Pathogenesis of glaucoma: integration of biomedical and Ayurvedic perspectives	no	study design / no quantitative data under the endpoints of interest
Bagnis	2011	128	Expert Opinion on Emerging Drugs	Current and emerging medical therapies in the treatment of glaucoma	no	study design / no quantitative data under the endpoints of interest
Park	2011	129	Korean J Ophthal	Short-Term Effects of Ginkgo biloba Extract on Peripapillary Retinal Blood Flow in Normal Tension Glaucoma	yes	
Cybulska-Heinrich	2012	130	Mol Vis	Ginkgo biloba: An adjuvant therapy for progressive normal and high tension glaucoma	no	no quantitative data under the endpoints of interest
Cimberle	2012	131	Ocular Surgery News	Lifestyle choices play important role in glaucoma management.	no	no quantitative data under the endpoints of interest
Milbury	2012	132	Journal of nutrition in gerontology a	Flavonoid intake and eye health	no	not on topic
Wang	2012	133	Sheng Li Xue Bao	[Ginkgolide B promotes axonal growth of retina ganglion cells by anti-apoptosis in vitro].	no	other (study design)
Mann	2012	134	Wiener Klinische Wochenschrift	Potentially inappropriate medication in geriatric patients: the Austrian consensus panel list.	no	other / not on topic
Alqawlaq	2012	135	Nanomedicine	Challenges in neuroprotective nanomedicine development: progress towards noninvasive gene therapy of glaucoma	no	other / not on topic
Cybulska-Heinrich	2012	136	Mol Vis	Ginkgo biloba: an adjuvant therapy for progressive normal and high tension glaucoma	no	study design / no quantitative data under the endpoints of interest
Baltmr	2012	137	Doctoral thesis	An investigation into pro-apoptotic targets in experimental glaucoma and the neuroprotective effects of Ginkgo biloba in retinal ganglion cells	no	study design / no quantitative data under the endpoints of interest
Zaghlool	2012	138	The Egyptian Journal of Histology	Histological and immunohistochemical study on the protective effect of Ginkgo biloba extract against glutamate-induced neurotoxicity in male alb	no	study design / no quantitative data under the endpoints of interest
Wang	2012	139	J Glaucoma	Survey of Complementary and Alternative Medicine Use in Glaucoma Patients	no	study design / no quantitative data under the endpoints of interest
Agrawal	2012	140	Clinical Medicine Reviews in Thera	Emerging Therapeutic Regimens for Glaucoma and Ocular Hypertension.	no	study design / no quantitative data under the endpoints of interest
Pascale	2012	141	Pharmacological Research	Protecting the retinal neurons from glaucoma: lowering ocular pressure is not enough	no	study design / no quantitative data under the endpoints of interest
Sosa	2012	142	Med Hypothesis Discov Innov Opht	Review on Hypothetical Implementing TGF-β Family Members in Glaucoma Therapy	no	study design / no quantitative data under the endpoints of interest
Osborne	2012	143	Ocular Blood Flow	Retinal Ischemia in Relation to Glaucoma and Neuroprotection	no	study design / no quantitative data under the endpoints of interest
Mckenna	2012	144	Botanical Medicines	Ginkgo Biloba	no	study design / no quantitative data under the endpoints of interest
Tataru	2012	145	Journal of medicine and life	Antiglaucoma pharmacotherapy	no	study design / no quantitative data under the endpoints of interest
Shim	2012	146	J Med Food	Ginkgo biloba extract and bilberry anthocyanins improve visual function in patients with normal tension glaucoma	yes	
Bromfield	2013	147	Current eye research	Use of complementary and alternative medicine for eye-related diseases and conditions	no	not on topic
McCarty	2013	148	Mitochondrial Physiology and Vege	The use of dietary supplements and their association with blood pressure in a large Midwestern cohort	no	not on topic
Hochwalt	2013	149	PLoS One	Ginkgo biloba Extract Individually Inhibits JNK Activation and Induces c-Jun Degradation in Human Chondrocytes: Potential Therapeutics for Osteo	no	other / not on topic
Inman	2013	150	PLoS One	α-Lipoic acid antioxidant treatment limits glaucoma-related retinal ganglion cell death and dysfunction	no	other / not on topic
Cybulska-Heinrich	2013	151	Klinische Monatsblätter für Augenh	[Value of non-IOP lowering therapy for glaucoma].	no	study design / no quantitative data under the endpoints of interest
Lee	2013	152	J Glaucoma	Effect of Ginkgo biloba extract on visual field progression in normal tension glaucoma	no	study design / no quantitative data under the endpoints of interest
Oh	2013	153	Current eye research	Effects of Ginkgo biloba Extract on Cultured Human Retinal Pigment Epithelial Cells under Chemical Hypoxia	no	study design / no quantitative data under the endpoints of interest
Schütt	2013	154	Ophthalmologe	Rolle des Energiestoffwechsels im retinalen Pigmentepithel	no	study design / no quantitative data under the endpoints of interest
Spadiene	2013	155	De Gruyter Open Access	Effect of ginkgo extract on eye microcirculation in patients with diabetes	no	study design / no quantitative data under the endpoints of interest
Mi	2013	156	Journal of Integrative Medicine	Research advances on the usage of traditional Chinese medicine for neuroprotection in glaucoma	no	study design / no quantitative data under the endpoints of interest
Lee	2013	157	J Glaucoma	Effect of Ginkgo biloba Extract on Visual Field Progression in Normal Tension Glaucoma	yes	
Jindal	2013	158	International Journal of Scientific and Research		no	other
Mozaffarieh	2013	159	Current opinion in pharmacology	New insights in the pathogenesis and treatment of normal tension glaucoma	no	other
Cybulska-Heinrich	2013	160	Klinische Monatsblätter für Augenh	Value of non-IOP lowering therapy for glaucoma	no	other
Aslan	2013	161	Redox Report	Oxidative stress and potential applications of free radical scavengers in glaucoma	no	study design / no quantitative data under the endpoints of interest
Mowatt	2013	162	Glaucoma-Basic and Clinical Aspe	Strategies for neuroprotection in glaucoma	no	study design / no quantitative data under the endpoints of interest
Chen	2013	163	Chinese medical journal	Neuroprotection in glaucoma: present and future	no	study design / no quantitative data under the endpoints of interest
Huynh	2013	164	Evidence-Based Complementary a	Botanical compounds: effects on major eye diseases	no	study design / no quantitative data under the endpoints of interest
Yoshida	2013	165	Journal of ocular pharmacology	Black currant anthocyanins normalized abnormal levels of serum concentrations of endothelin-1 in patients with glaucoma	no	not on topic
Sabater-Jara	2013	166	Phytochemistry Reviews	Biotechnological approaches to enhance the biosynthesis of ginkgolides and bilobalide in Ginkgo biloba	no	not on topic
Lee	2014	167	J Korean Ophthalmol Soc	The Effect of Anthocyanoside and Ginkgo Biloba Extract on Normal-Tension Glaucoma According to Presence of Diabetes	no	Different supplements
Droy-Lefaix	2014	168	Les Cahiers d'Ophtalmologie	Citicoline, Ginkgo biloba et magnésium dans le glaucome	no	Different supplements
Dautova	2014	169	Vestn Oftalmol.	[Clinical efficacy of Vitrum Memory in patients with glaucoma and dry form of age-related macular degeneration]	no	Language incompatibility
Nuhu	2014	170	Journal of Applied Pharmaceutical	Ginkgo biloba: A 'living fossil'with modern day phytomedicinal applications	no	no quantitative data under the endpoints of interest
Grover	2014	171	Molecular and Cellular Biochemist	Antioxidants and vision health: facts and fiction	no	not on topic
Komori	2014	172	Graefe's Archive for Clinical and Ex	Results of long-term monitoring of normal-tension glaucoma patients receiving medical therapy: results of an 18-year follow-up	no	not on topic
Ekici	2014	173	International Scholarly Research N	The role of magnesium in the pathogenesis and treatment of glaucoma	no	other / not on topic
Osborne	2014	174	Drug discovery today	Targeting mitochondrial dysfunction as in aging and glaucoma	no	other / not on topic
Malishevskaia	2014	175	Vestn Oftalmol.	[Options for correction of endothelial dysfunction and oxidative stress in patients with primary open-angle glaucoma].	no	other / not on topic / language incompatibility
Guo	2014	176	IOVS	Effect of Ginkgo biloba on visual field and contrast sensitivity in Chinese patients with normal tension glaucoma: a randomized, crossover clinical t	no	study design / no quantitative data under the endpoints of interest
James	2014	177	Current Medical Literature: Ophtha	Intracameral bevacizumab as an adjunct to trabeculectomy: a 1-year prospective, randomised study.	no	study design / no quantitative data under the endpoints of interest

Guo	2014	178	IOVS	Author response: Ginkgo biloba extract improves visual field damage in some patients affected by normal-tension glaucoma	no	study design / no quantitative data under the endpoints of interest
Song	2014	179	Indian journal of ophthalmology	New directions in the treatment of normal tension glaucoma	no	study design / no quantitative data under the endpoints of interest
Bhartiya	2014	180	Journal of Current Glaucoma Practi	Complementary and alternate management of glaucoma: The verdict so far	no	study design / no quantitative data under the endpoints of interest
Pandey	2014	181	Portal Regional de BVS	Glaucoma: role of neuroprotective agents.	no	study design / no quantitative data under the endpoints of interest
Jabbarpoor Bonyadi	2014	182	BMC Complementary and Alternati	The ocular hypotensive effect of saffron extract in primary open angle glaucoma: a pilot study	no	study design / no quantitative data under the endpoints of interest
Mohanta	2014	183	Natural product research	Phytochemical and medicinal importance of Ginkgo biloba L.	no	study design / no quantitative data under the endpoints of interest
James	2014	184	Current Medical Literature: Ophtha	Effect of ginkgo biloba on visual field and contrast sensitivity in Chinese patients with normal tension glaucoma: a randomized, crossover clinical tr	no	study design / no quantitative data under the endpoints of interest
Bower	2014	185	J Glaucoma	Canadian ophthalmologists' opinions concerning complementary and alternative medicine (CAM) use in glaucoma	no	study design / no quantitative data under the endpoints of interest
Stanford	2014	186	Ophthalmic Practice	Traditional Chinese medicine, health beliefs and glaucoma awareness: implications for UK practice	no	study design / no quantitative data under the endpoints of interest
Khalil	2014	187	International Ophthalmology Clinic	Medical Management of Exfoliative Glaucoma	no	study design / no quantitative data under the endpoints of interest
Pescosolido	2014	188	Advances in Ophthalmology & Visu	Critical review on the relationship between glaucoma and alzheimer's disease	no	study design / no quantitative data under the endpoints of interest
Chu	2014	189	Taiwan Journal of Ophthalmology	Herbal molecules in eye diseases	no	study design / no quantitative data under the endpoints of interest
Anand	2014	190	Journal of Cellular Biochemistry	Preserving Neural Retina Through Re-Emerging Herbal Interventions	no	study design / no quantitative data under the endpoints of interest
Guo	2014	191	IOVS	Effect of Ginkgo Biloba on Visual Field and Contrast Sensitivity in Chinese Patients With Normal Tension Glaucoma: A Randomized, Crossover Clini	yes	
Garcia-Medina	2015	192	Acta Ophthalmologica	A two-year follow-up of oral antioxidant supplementation in primary open-angle glaucoma: an open-label, randomized, controlled trial	no	Different supplements
Brodie	2015	193	IOVS	Eyedrop Formulation and Evaluation of Quercetin-a component of Ginkgo biloba	no	Different supplements
Cao	2015	194	Current Eye Research	Prevention of Selenite-Induced Cataratogenesis by Ginkgo biloba Extract (Egb761) in Wistar Rats	no	other / not on topic
Töteberg-Harms	2015	195	Der Ophthalmologe	Current aspects on the management of normal tension glaucoma	no	other / not on topic
Milanez	2015	196	African Journal of Pharmacy and Ph	Ginkgo bilobaL.: Phytochemical components and antioxidant activity	no	other / not on topic
Fang	2015	197	Expert Review of Ophthalmology	The effect of Ginkgo biloba and Nifedipine on DNA breaks in circulating leukocytes of glaucoma patients	no	study design / no quantitative data under the endpoints of interest
Tian	2015	198	Clinical Ophthalmology	Current perspective of neuroprotection and glaucoma	no	study design / no quantitative data under the endpoints of interest
Fermino	2015	199	African Journal of Pharmacy and Ph	Ginkgo biloba L.: Phytochemical components and antioxidant activity	no	study design / no quantitative data under the endpoints of interest
Nash	2015	200	Integrative medicine insights	Current Perspectives on the Beneficial Role of Ginkgo biloba in Neurological and Cerebrovascular Disorders	no	study design / no quantitative data under the endpoints of interest
Pinazo-Duran	2015	201	Progress in Brain Research	Oxidative stress and mitochondrial failure in the pathogenesis of glaucoma neurodegeneration	no	study design / no quantitative data under the endpoints of interest
Song	2015	202	Drug Design, Development and The	Neuroprotective therapies for glaucoma	no	study design / no quantitative data under the endpoints of interest
Patel	2015	203	Graefe's Archive for Clinical and Ex	The effect of flavonoids on visual function in patients with glaucoma or ocular hypertension: a systematic review and meta-analysis	no	study design / no quantitative data under the endpoints of interest
Ciotu	2015	204	J Med Life	Biochemical changes and treatment in glaucoma	no	study design / no quantitative data under the endpoints of interest
Reed	2015	205	Review of Optometry	Nutrition and glaucoma: exploring the link: numerous studies reveal that nutrition can play a big role in glaucoma development and management	no	study design / no quantitative data under the endpoints of interest
Barbosa-Breda	2015	206	Revista Sociedade	Advances in glaucoma pharmacological therapeutics	no	study design / no quantitative data under the endpoints of interest
Wu	2015	207	Urology	Effect of Ginkgo biloba extract (EGb-761) on recovery of erectile dysfunction in bilateral cavernous nerve injury rat model	no	study design / no quantitative data under the endpoints of interest
Mastropasqua	2015	208	Progress in Brain Research	Advance in the pathogenesis and treatment of normal-tension glaucoma	no	study design / no quantitative data under the endpoints of interest
Koo	2015	209	European Archives of Oto-Rhino-La	The efficacy and safety of systemic injection of Ginkgo biloba extract, EGb761, in idiopathic sudden sensorineural hearing loss: a randomized place	no	study design / no quantitative data under the endpoints of interest
Martínez-Domínguez	2015	210	Microchemical Journal	Determination of toxic substances, pesticides and mycotoxins, in ginkgo biloba nutraceutical products by liquid chromatography Orbitrap-mass sp	no	study design / no quantitative data under the endpoints of interest
Morrone	2015	211	Progress in Brain Research	Natural compounds and retinal ganglion cell neuroprotection	no	study design / no quantitative data under the endpoints of interest
Jung	2016	212	The Journal of Korean Medicine Opl	The Study on the Korean and Western Medical Literatures for Neuroprotection Therapy of Glaucoma	no	Language incompatibility
Doozandeh	2016	213	Journal of ophthalmic & vision rese	Neuroprotection in glaucoma	no	study design / no quantitative data under the endpoints of interest
Ferreira	2016	214	repositorio	Normal tension glaucoma: literature review	no	study design / no quantitative data under the endpoints of interest
Esporcatte	2016	215	Arquivos brasileiros de oftalmologi	Normal-tension glaucoma: an update	no	study design / no quantitative data under the endpoints of interest
Gossman	2016	216	Current pharmaceutical design	Neuroprotective strategies in glaucoma	no	study design / no quantitative data under the endpoints of interest
Gauthier	2016	217	The Yale journal of biology and mec	Focus: the aging brain: neurodegeneration and neuroprotection in glaucoma	no	study design / no quantitative data under the endpoints of interest
Chen	2016	218	IOVS	The association between glaucoma and chocolate consumption	no	study design / no quantitative data under the endpoints of interest
Pasquale	2016	219	Pearls of Glaucoma Management	Medical Treatment: Alternative Medicine and Glaucoma	no	study design / no quantitative data under the endpoints of interest
Yang	2016	220	IOVS	Antioxidant treatment limits neuroinflammation in experimental glaucoma	no	study design / no quantitative data under the endpoints of interest
Esporcatte	2016	221	Arquivos brasileiros de oftalmologi	Glaucoma de pressão normal: atualização	no	study design / no quantitative data under the endpoints of interest
Ergan	2016	222	Int J Ophthalmol	Oxidant/antioxidant balance in the aqueous humor of patients with glaucoma	no	study design / no quantitative data under the endpoints of interest
Wojcik-Gryciuk	2016	223	Restorative Neurology and Neurosc	Glaucoma–state of the art and perspectives on treatment	no	study design / no quantitative data under the endpoints of interest
Mikheytseva	2016	224	Ukrainian biopharmaceutical journ	Oxidative stress correction with metabolic preparations, new possibilities of antioxidant protection in primary glaucoma	no	study design / no quantitative data under the endpoints of interest
Sari	2016	225	International Journal of PharmTech	Ginkgo Biloba Extract Effect on Oxidative Stress Marker Malonildialdehyde, Redox Enzyme Gluthation Peroxidase, Visual Field Damage, and Retinal	yes	
Harris	2017	226	Acta Ophthalmologica	The effects of antioxidants on ocular blood flow in patients with glaucoma	no	Different supplements
Nuzzi	2017	227	Frontiers in Neuroscience	Glaucoma: biological trabecular and neuroretinal pathology with perspectives of therapy innovation and preventive diagnosis	no	not on topic
Asano	2017	228	Scientific reports	Age-and sex-dependency of the association between systemic antioxidant potential and glaucomatous damage	no	other / not on topic
Vahedian	2017	229	EPMA Journal	Nutritional recommendations for individuals with Flammer syndrome	no	study design / no quantitative data under the endpoints of interest
Rusciano	2017	230	Advances in Pharmacological and F	Neuroprotection in glaucoma: Old and new promising treatments	no	study design / no quantitative data under the endpoints of interest
Graubard	2017	231	Glaucomatoday	Complementary and Alternative Medicine for Glaucoma	no	study design / no quantitative data under the endpoints of interest
Han	2017	232	Journal of Zhejiang Chinese Medica	Study on the Effect of Ginkgo Biloba Extract on Optic Nerve in Progressive Normal-tension Glaucoma Patients / 浙江中医药大学学报	no	study design / no quantitative data under the endpoints of interest
Andreeva	2017	233	Varna Medical Forum	Pharmacological activity of ginkgo biloba in the treatment of glaucoma	no	study design / no quantitative data under the endpoints of interest
Lin	2017	234	digitalnz.org	Protective effect of nerve growth factor associated with ginkgo biloba extraction on acute glaucoma retinal ischemia reperfusion injury in rabbit	no	study design / no quantitative data under the endpoints of interest
Sena	2017	235	Cochrane Database of Systematic I	Neuroprotection for treatment of glaucoma in adults	no	study design / no quantitative data under the endpoints of interest
Khan	2017	236	Research and Therapy	Molecular basis of glaucoma and its therapeutical analysis in Pakistan: an overview	no	study design / no quantitative data under the endpoints of interest
Turner	2017	237	Current Ophthalmology Reports	Diagnosis and Monitoring of Low-Tension Glaucoma	no	study design / no quantitative data under the endpoints of interest

Yue-Mei	2017	238	Portal Regional da BVS	Protective effect of nerve growth factor associated with ginkgo biloba extraction on acute glaucoma retinal ischemia reperfusion injury in rabbit	no	study design / no quantitative data under the endpoints of interest
Wang	2017	239	Journal of Pharmacy	Pharmacokinetic and ocular microdialysis study of oral ginkgo biloba extract in rabbits by UPLC-MS/MS determination	no	study design / no quantitative data under the endpoints of interest
Konieczka	2017	240	Klinische Monatsblätter für Augenh	Leber's Hereditary Optic Neuropathy, Normal Tension Glaucoma, and Flammer Syndrome: Long Term Follow-up of a Patient	no	study design / no quantitative data under the endpoints of interest
Cohen	2017	241	The Journal of Clinical and Aestheti	Fixed drug eruption to supplement containing Ginkgo biloba and vinpocetine: a case report and review of related cutaneous side effects	no	study design / no quantitative data under the endpoints of interest
Toris	2017	242	Journal of Ocular Pharmacology an	Making basic science studies in glaucoma more clinically relevant: the need for a consensus	no	study design / no quantitative data under the endpoints of interest
Zhou	2017	243	Biomedicine & Pharmacotherapy	Long-term pre-treatment of antioxidant Ginkgo biloba extract EGb-761 attenuates cerebral-ischemia-induced neuronal damage in aged mice	no	study design / no quantitative data under the endpoints of interest
Symes	2017	244	Canadian Journal of Ophthalmolog	Normal tension glaucoma management: a survey of contemporary practice	no	study design / no quantitative data under the endpoints of interest
Kang	2018	245	Acta Ophthalmologica	Prospective study of flavonoid intake and risk of primary open-angle glaucoma	no	Different supplements
Kang	2018	246	Current opinion in ophthalmology	Ginkgo biloba and its potential role in glaucoma	no	no quantitative data under the endpoints of interest
Parisi	2018	247	Current Neuropharmacology	Citicoline and retinal ganglion cells: effects on morphology and function	no	not on topic
Rapino	2018	248	Current Neuropharmacology	Neuroprotection by (endo) cannabinoids in glaucoma and retinal neurodegenerative diseases	no	other / not on topic
Guzun	2018	249	Journal of Ophthalmology (Ukraine)	Efficacy of complex neuroprotection in glaucomatous optic neuropathy	no	other / not on topic
Pinazo-Duran	2018	250	Current Neuropharmacology	Strategies to reduce oxidative stress in glaucoma patients	no	study design / no quantitative data under the endpoints of interest
Kang	2018	251	Current opinion in ophthalmology	Ginkgo biloba and its potential role in glaucoma	no	study design / no quantitative data under the endpoints of interest
Antonio	2018	252	Current Neuropharmacology	Rational basis for nutraceuticals in the treatment of glaucoma	no	study design / no quantitative data under the endpoints of interest
Owaifeer	2018	253	Ophthalmology and therapy	The role of diet in glaucoma: a review of the current evidence	no	study design / no quantitative data under the endpoints of interest
Anton-Lopez	2018	254	Archivos de la Sociedad Española d	ifestyles guide and glaucoma (II). Diet, supplements, drugs, sleep, pregnancy, and systemic hypertension	no	study design / no quantitative data under the endpoints of interest
Nucci	2018	255	Eye	Neuroprotective agents in the management of glaucoma	no	study design / no quantitative data under the endpoints of interest
Rohini	2018	256	Drug Invention Today	Natural remedies for management of glaucoma	no	study design / no quantitative data under the endpoints of interest
Ramdas	2018	257	Acta ophthalmologica	The relation between dietary intake and glaucoma: a systematic review	no	study design / no quantitative data under the endpoints of interest
Adeghate	2018	258	Survey of ophthalmology	Intraocular pressure–independent management of normal tension glaucoma	no	study design / no quantitative data under the endpoints of interest
He	2018	259	Journal of Ocular Pharmacology an	Targets of Neuroprotection in Glaucoma	no	study design / no quantitative data under the endpoints of interest
Hochwalt	2018	260	Review of Optometry	GLAUCOMA: LIFESTYLES OF THE ANTIOXIDANT RICH AND FAMOUS	no	study design / no quantitative data under the endpoints of interest
Singh	2018	261	Ophthalmology Times	Looking beyond IOP when managing glaucoma patients: Nutrition is a potentially preventative therapy that should be supplementary to treatment	no	study design / no quantitative data under the endpoints of interest
McMonnies	2018	262	Journal of optometry	Reactive oxygen species, oxidative stress, glaucoma and hyperbaric oxygen therapy	no	study design / no quantitative data under the endpoints of interest
Sigireddi	2018	263	International Ophthalmology Clinic	Neuroprotection in glaucoma	no	study design / no quantitative data under the endpoints of interest
Yang	2018	264	Medicine	Evidence-based practice guideline of Chinese herbal medicine for primary open-angle glaucoma (qingfeng -neizhang)	no	study design / no quantitative data under the endpoints of interest
Rajarajan	2018	265	Drug Invention Today	A review on the medicinal properties of Gingko biloba.	no	study design / no quantitative data under the endpoints of interest
Aziz	2018	266	Therapeutics and Clinical Risk Man	Efficacy and safety of Ginkgo biloba extract as an “add-on” treatment to metformin for patients with metabolic syndrome: a pilot clinical study	no	study design / no quantitative data under the endpoints of interest
Huang	2018	267	Frontiers in Medicine	Asiatic acid prevents retinal ganglion cell apoptosis in a rat model of glaucoma	no	study design / no quantitative data under the endpoints of interest
Sacca	2019	268	Nutrients	Substances of interest that support glaucoma therapy	no	Different supplements
Martínez-Solís	2019	269	Planta Med	Neuroprotective potential of Ginkgo biloba in retinal diseases	no	study design / no quantitative data under the endpoints of interest
Cho	2019	270	Journal of medicinal food	Neuroprotective Effect of Ginkgo Biloba Extract Against Hypoxic Retinal Ganglion Cell Degeneration In Vitro and In Vivo	no	study design / no quantitative data under the endpoints of interest
Loskutova	2019	271	Survey of ophthalmology	Nutritional supplementation in the treatment of glaucoma: A systematic review	no	study design / no quantitative data under the endpoints of interest
Zueva	2019	272	Neural Networks and Neurotechno	Non-pharmacological methods of neuroprotection and neurorehabilitation	no	study design / no quantitative data under the endpoints of interest
Kwong	2019	273	Medical Treatment of Glaucoma	Novel Therapeutic Targets for Glaucoma: Disease Modification Treatment, Neuroprotection, and Neuroregeneration	no	study design / no quantitative data under the endpoints of interest
Sherif	2019	274	Biomolecules	Ginkgo Biloba Extract Alleviates Methotrexate-Induced Renal Injury: New Impact on PI3K/Akt/mTOR Signaling and MALAT1 Expression	no	study design / no quantitative data under the endpoints of interest
Fu	2019	275	BioMed Research International	Therapeutic Strategies for Attenuation of Retinal Ganglion Cell Injury in Optic Neuropathies: Concepts in Translational Research and Therapeutic In	no	study design / no quantitative data under the endpoints of interest
Suvarna	2019	276	Handbook of Nutrition, Diet, and th	Role of Natural Products in Glaucoma Management	no	study design / no quantitative data under the endpoints of interest
Avetisov	2019	277	J Glaucoma	Rationale for neuroprotection in glaucoma	no	study design / no quantitative data under the endpoints of interest
Adornetto	2019	278	Neural regeneration research	Neuroinflammation as a target for glaucoma therapy	no	study design / no quantitative data under the endpoints of interest
Ongkeko-Perez	2019	279	Philipp J Ophthalmol	Do you give neuroprotective/alternative drugs to manage normal tension glaucoma?	no	study design / no quantitative data under the endpoints of interest
Cocco	2019	280	Minerva Oftalmologica	Diet and glaucoma	no	study design / no quantitative data under the endpoints of interest
Yang	2019	281	Journal of current glaucoma practic	Drug-induced acute angle-closure glaucoma: a review	no	study design / no quantitative data under the endpoints of interest
Zhang	2019	282	Neuropsychiatric Disease and Trea	Ginkgo biloba Extract Reduces Hippocampus Inflammatory Responses, Improves Cardiac Functions And Depressive Behaviors In A Heart Failure M	no	study design / no quantitative data under the endpoints of interest
Sacca	2019	283	Handbook of Nutrition, Diet, and th	Glaucoma: an overview	no	study design / no quantitative data under the endpoints of interest
Menezes	2019	284	Glaucoma	What's New in Alternative Therapies for Glaucoma	no	study design / no quantitative data under the endpoints of interest
Perez	2019	285	Current Opinion in Ophthalmology	Relationship of lifestyle, exercise, and nutrition with glaucoma	no	study design / no quantitative data under the endpoints of interest
Guglielmi	2019	286	Expert Opinion on Therapeutic Pate	Novel therapies for glaucoma: a patent review (2013-2019)	no	study design / no quantitative data under the endpoints of interest
Bungau	2019	287	Oxidative Medicine and Cellular Lo	Health benefits of polyphenols and carotenoids in age-related eye diseases	no	study design / no quantitative data under the endpoints of interest
Lawler	2019	288	Nutrients	Dietary antioxidants, macular pigment, and glaucomatous neurodegeneration: a review of the evidence	no	study design / no quantitative data under the endpoints of interest
Silva	2019	289	Plants	Ginkgo biloba L. Leaf Extract Protects HepG2 Cells Against Paraquat-Induced Oxidative DNA Damage	no	study design / no quantitative data under the endpoints of interest
Oliveira	2019	290	European Journal of Nutition	Antioxidant and antigenotoxic activities of Ginkgo biloba L. leaf extract are retained after in vitro gastrointestinal digestive conditions	no	study design / no quantitative data under the endpoints of interest
Labkovich	2020	291	Asia-Pacific Journal of Ophthalmolo	Ginkgo Biloba Extract in Ophthalmic and Systemic Disease, With a Focus on Normal-Tension Glaucoma	no	no quantitative data under the endpoints of interest
Sen	2020	292	Eye	Neurodegeneration in Alzheimer's disease and glaucoma: overlaps and missing links	no	no quantitative data under the endpoints of interest
Peter	2020	293	Advanced Pharmaceutical Bulletin	A review on newer ocular drug delivery systems with an emphasis on glaucoma	no	other / not on topic
Rossetti	2020	294	J Glaucoma	Can treatment with citicoline eyedrops reduce progression in glaucoma? The results of a randomized placebo-controlled clinical trial	no	other / not on topic
Rahic	2020	295	Pharmaceuticals	Novel drug delivery systems fighting glaucoma: Formulation obstacles and solutions	no	other / not on topic
Labkovich	2020	296	Asia Pac J Ophthalmol (Phila)	Ginkgo Biloba Extract in Ophthalmic and Systemic Disease, With a Focus on Normal-Tension Glaucoma	no	study design / no quantitative data under the endpoints of interest
Ige	2020	297	The Yale Journal of Biology and Mec	Herbal Medicines in Glaucoma Treatment	no	study design / no quantitative data under the endpoints of interest

Garcia-Medina	2020	298	Antioxidants	Glaucoma and antioxidants: Review and update	no	study design / no quantitative data under the endpoints of interest
Emrani	2020	299	Augenheilkunde up2date	Medikamentöse Therapie des Glaukoms	no	study design / no quantitative data under the endpoints of interest
Fei	2020	300	Neural Regeneration Research	Neuroprotection mediated by natural products and their chemical derivatives	no	study design / no quantitative data under the endpoints of interest
Adornetto	2020	301	Nutrients	Natural products: evidence for neuroprotection to be exploited in glaucoma	no	study design / no quantitative data under the endpoints of interest
Wen	2020	302	TMR Modern Herbal Medicine	Effects of Ginkgo biloba extract on diabetic retinopathy: A meta-analysis and systematic review	no	study design / no quantitative data under the endpoints of interest
Fang	2020	303	OBM Neurobiology	Neuroprotective strategies in glaucoma-translation to clinical trials	no	study design / no quantitative data under the endpoints of interest
Jabbehdari	2020	304	European Journal of Ophthalmolog	Effect of dietary modification and antioxidant supplementation on intraocular pressure and open-angle glaucoma	no	study design / no quantitative data under the endpoints of interest
Charters	2020	305	Ophthalmologytimes	Glaucoma treatment alternatives Thinking outside the box	no	study design / no quantitative data under the endpoints of interest
Chen	2020	306	Taiwan Journal of Ophthalmology	Normal tension glaucoma in Asia: Epidemiology, pathogenesis, diagnosis, and management	no	study design / no quantitative data under the endpoints of interest
Emrani	2020	307	Klinische Monatsblätter für Augenh	Medical management of Glaucoma	no	study design / no quantitative data under the endpoints of interest
De Souza	2020	308	Planta Medica	Effects of Ginkgo biloba on diseases related to oxidative stress	no	study design / no quantitative data under the endpoints of interest
Cvenkel	2020	309	Journal of Ophthalmology	Current medical therapy and future trends in the management of glaucoma treatment	no	study design / no quantitative data under the endpoints of interest
Han	2020	310	Korean J Ophthal	Normal-tension glaucoma management: a survey of glaucoma sub-specialists in Korea	no	study design / no quantitative data under the endpoints of interest
Bhartiya	2020	311	Clinical and Experimental Vision ar	Is it time for Precision Medicine in Glaucoma?	no	study design / no quantitative data under the endpoints of interest
Zarei	2020	312	Austin Ophthalmology	Updates on Normal Tension Glaucoma Management	no	study design / no quantitative data under the endpoints of interest
Himori	2020	313	Researchsquare	The Effect of Dietary Antioxidant Supplementation in Patients with Cataract and Glaucoma	no	study design / no quantitative data under the endpoints of interest
Yadav	2020	314	Life sciences	Bio-tactics for neuroprotection of retinal ganglion cells in the treatment of glaucoma	no	study design / no quantitative data under the endpoints of interest
Chen	2020	315	Taiwan Journal of Ophthalmology	Is intraocular pressure reduction the key treatment for normal-tension glaucoma?	no	study design / no quantitative data under the endpoints of interest
Naik	2020	316	European Journal of Pharmacology	Neuroprotection: A versatile approach to combat glaucoma	no	study design / no quantitative data under the endpoints of interest
Ekici-Günay	2020	317	Pathology	Ginkgo biloba extract as an antioxidant in nerve regeneration	no	study design / no quantitative data under the endpoints of interest
Jonas	2020	318	The Asia-Pacific Journal of Ophthal	Advances and latest developments in ophthalmology and visual sciences	no	study design / no quantitative data under the endpoints of interest
Behtaj	2020	319	Tissue Engineering and Regenerativ	Retinal tissue bioengineering, materials and methods for the treatment of glaucoma	no	study design / no quantitative data under the endpoints of interest
Gonzalez Fleitas	2020	320	Journal of Neurochemistry	Enriched environment provides neuroprotection against experimental glaucoma	no	study design / no quantitative data under the endpoints of interest
Coyle	2021	321	Biomolecules	Targeting the NLRP3 inflammasome in glaucoma	no	not on topic
Kangilbaeva	2021	322	International Journal of Pharmaceu	Effect of EGb 761 (tanakan) therapy in eyes with nonproliferative diabetic retinopathy	no	not on topic
Päärmann	2021	323	Deutsche Zeitschrift für Akupunktu	Alternative und integrative Medizin (AIM) bei verschiedenen Glaukomformen	no	other / not on topic
Rahic	2021	324	Kompass der Ophthalmologie	Neuartige Wirkstoffabgabesysteme für die Glaukomtherapie: Hürden und Lösungsansätze für die Formulierung	no	other / not on topic
Tribble	2021	325	Cells	Targeting diet and exercise for neuroprotection and neurorecovery in glaucoma	no	other / not on topic
Zhu	2021	326	Frontiers in Medicine	Oral scutellarin treatment ameliorates retinal thinning and visual deficits in experimental glaucoma	no	other / not on topic
Fan Gaskin	2021	327	Antioxidants	Oxidative stress and the role of NADPH oxidase in glaucoma	no	other / not on topic
Barth	2021	328	Molecular and Cellular Neuroscien	Pharmacologic treatments in preclinical tinnitus models with special focus on Ginkgo biloba leaf extract EGb 761®	no	other / not on topic
Sun	2021	329	American Journal of Otolaryngology	Effectiveness of Ginkgo biloba diterpene lactone in the treatment of sudden sensorineural hearing loss	no	other / not on topic
Roychoudhury	2021	330	Herbal Medicine in Andrology	Herbal medicine use to treat andrological problems: Asian and Indian subcontinent: Ginkgo biloba, Curcuma longa, and Camellia sinensis	no	other / not on topic
Park	2021	331	PLoS One	Relationship between peripheral vasospasm and visual field progression rates in patients with normal-tension glaucoma with low-teen intraocular	no	other / not on topic
Pillunat	2021	332	Der Ophthalmologe	Vasculat treatment concepts in glaucoma patients	no	study design / no quantitative data under the endpoints of interest
Li	2021	333	Experimental Eye Research	Procyanidin B2 and rutin in Ginkgo biloba extracts protect human retinal pigment epithelial (RPE) cells from oxidative stress by modulating Nrf2 and	no	study design / no quantitative data under the endpoints of interest
Plummer	2021	334	Veterinary Ophthalmology	Prophylactic anti-glaucoma therapy in dogs with primary glaucoma: A practitioner survey of current medical protocols	no	study design / no quantitative data under the endpoints of interest
Shen	2021	335	Experimental Eye Research	Protection of retinal ganglion cells in glaucoma: Current status and future	no	study design / no quantitative data under the endpoints of interest
Ng	2021	336	European Journal of Integrative Mec	Complementary and alternative medicine mentions and recommendations in glaucoma guidelines: Systematic review and quality assessment	no	study design / no quantitative data under the endpoints of interest
Himori	2021	337	Clinical Ophthalmology	The Effect of Dietary Antioxidant Supplementation in Patients with Glaucoma	no	study design / no quantitative data under the endpoints of interest
More	2021	338	GSC Biological and Pharmaceutica	Pharmacognosy, phytochemistry, pharmacology and clinical application of Ginkgo biloba	no	study design / no quantitative data under the endpoints of interest
Chudhary	2021	339	Experimental and Therapeutic Med	Ginkgo biloba delays light-induced photoreceptor degeneration through antioxidant and antiapoptotic properties	no	study design / no quantitative data under the endpoints of interest
Kulathunga	2021	340	The Journal of the College of Ophth	Latest developments in glaucoma	no	study design / no quantitative data under the endpoints of interest
Storgaard	2021	341	Frontiers in Medicine	Glaucoma clinical research: Trends in treatment strategies and drug development	no	study design / no quantitative data under the endpoints of interest
Ke	2021	342	Drug Design, Development and The	The Synergistic Effect of Ginkgo biloba Extract 50 and Aspirin Against Platelet Aggregation	no	study design / no quantitative data under the endpoints of interest
Arrigo	2021	343	Frontiers in Aging	Cognitive dysfunctions in glaucoma: an overview of morpho-functional mechanisms and the impact on higher-order visual function	no	study design / no quantitative data under the endpoints of interest
Konieczka	2021	344	Journal of Clinical Medicine	Treatment of glaucoma patients with flammer syndrome	no	study design / no quantitative data under the endpoints of interest
Yang	2021	345	Chinese Medicine	Integrating network pharmacological and experimental models to investigate the therapeutic effects of baicalein in glaucoma	no	study design / no quantitative data under the endpoints of interest
Sabaner	2021	346	International Ophthalmology	Ginkgo Biloba affects microvascular morphology: a prospective optical coherence tomography angiography pilot study	yes	
Anton	2022	347	Life	The Effect of oral citicoline and docosahexaenoic acid on the visual field of patients with glaucoma: A randomized trial	no	Different supplements
Bakunina	2022	348	Russian Ophthalmological Journal	The life style of glaucoma patients: a scientific rationale for basic recommendations	no	no quantitative data under the endpoints of interest
Nafees	2022	349	Avicenna journal of phytomedicine	Indian traditional medicinal plants in ophthalmic diseases	no	no quantitative data under the endpoints of interest
Mlynarczyk	2022	350	Nutrients	Diet, oxidative stress, and blood serum nutrients in various types of glaucoma: a systematic review	no	not on topic
Wolfram	2022	351	Die Ophthalmologie	Glaukomversorgung in Deutschland–Ergebnisse einer Mitglieder-Umfrage von DOG und BVA: Teil 2: Therapie	no	other / not on topic
Billeit	2022	352	Präventionsmedizin und Anti-Aging	Ophthalmologisches Altern	no	other / not on topic
Prokosch	2022	353	Die Ophthalmologie	Regeneration des Sehnerven–Wird das einmal Realität?	no	other / not on topic
Shelestun	2022	354	Journal of Healthy Nutrition and Die	Ginkgo biloba–proven benefits and how to apply	no	other / not on topic
Türksever	2022	355	Metabolites	Peripapillary Oxygenation and Retinal Vascular Responsiveness to Flicker Light in Primary Open Angle Glaucoma	no	other / not on topic
Elwahidy	2022	356	Al-Azhar International Medical Jour	Gingko Biloba as an adjuvant to Timolol in Moderate Primary Open Angle Glaucoma	no	other / not on topic
Sim	2022	357	Nutients	Treatment of glaucoma with natural products and their mechanism of action: An update	no	study design / no quantitative data under the endpoints of interest

Hua	2022	358	Chinese Journal of Natural Medicin	A network pharmacology-based strategy for predicting the protective mechanism of Ginkgo biloba on damaged retinal ganglion cells	no	study design / no quantitative data under the endpoints of interest
Chaudhry	2022	359	Survey of ophthalmology	Nutritional supplementation in the prevention and treatment of glaucoma	no	study design / no quantitative data under the endpoints of interest
Vishwaraj	2022	360	Indian Journal of Ophthalmology	Neuroprotection in glaucoma	no	study design / no quantitative data under the endpoints of interest
Tang	2022	361	Glaucoma - Recent Advances and I	The molecular mechanisms of trabecular meshwork damage in POAG and treatment advances	no	study design / no quantitative data under the endpoints of interest
Hurley	2022	362	Antioxidants	The intertwined roles of oxidative stress and endoplasmic reticulum stress in glaucoma	no	study design / no quantitative data under the endpoints of interest
Li	2022	363	World Journal of Traditional Chines	Andrographolide Protects Retinal Ganglion Cells in Rats with Glaucoma by Regulating the Bcl-2/Bax/caspase-3 Signaling Pathway	no	study design / no quantitative data under the endpoints of interest
Mohan	2022	364	Indian Journal of Ophthalmology	Newer advances in medical management of glaucoma	no	study design / no quantitative data under the endpoints of interest
Palakkamanil	2022	365	IOVS	The Effect of Gingko Biloba Extract (GBE) on Optic Nerve Head Perfusion Examined Using Ocular Coherence Tomography Angiography (OCT-A): A P	no	study design / no quantitative data under the endpoints of interest
Kuo	2022	366	Journal of Personalized Medicine	Neuroprotection in glaucoma: basic aspects and clinical relevance	no	study design / no quantitative data under the endpoints of interest
Fahmideh	2022	367	Survey of ophthalmology	Non-drug interventions in glaucoma: Putative roles for lifestyle, diet and nutritional supplements	no	study design / no quantitative data under the endpoints of interest
Ahmad	2022	368	touchOphthalmology	Dietary Intervention in Glaucoma	no	study design / no quantitative data under the endpoints of interest
Ramírez-González	2022	369	Ciencia y Tecnolgia para la Salud V	El extracto de Ginkgo Biloba 761 reduce el riesgo de la progresión de la generación macular asociada a la edad	no	study design / no quantitative data under the endpoints of interest
Silva	2022	370	Biology	Cardiovascular activity of Ginkgo biloba—An insight from healthy subjects	no	study design / no quantitative data under the endpoints of interest
Caceres-Velez	2022	371	Ageing Research	Restoring the oxidative balance in age-related diseases–An approach in glaucoma	no	study design / no quantitative data under the endpoints of interest
Casson	2022	372	Clinical & Experimental Ophthalmo	Medical therapy for glaucoma: A review	no	study design / no quantitative data under the endpoints of interest
Młynarczyk	2022	373	Nutrients	Diet, oxidative stress, and blood serum nutrients in various types of glaucoma: a systematic review	no	study design / no quantitative data under the endpoints of interest
Leung	2022	374	Clinical & experimental ophthalmo	Normal-tension glaucoma: current concepts and approaches-a review	no	study design / no quantitative data under the endpoints of interest
Lambuk	2022	375	Eye and Vision	Nanoparticles for the treatment of glaucoma-associated neuroinflammation	no	study design / no quantitative data under the endpoints of interest
Wu	2022	376	Practice and Research	Role of ocular blood flow in normal tension glaucoma	no	study design / no quantitative data under the endpoints of interest
Sim	2022	377	Nutrients	Treatment of Glaucoma with Natural Products and Their Mechanism of Action: An Update. Nutrients 2022, 14, 534	no	study design / no quantitative data under the endpoints of interest
Tsigkos	2022	378	Acta Ophthalmologica	“The glaucoma NO-ctail” the use of vasodilators and OCT-A in ischemic optic-nerve: Case report	no	study design / no quantitative data under the endpoints of interest
Saha	2022	379	Kerala Journal of Ophthalmology	Lifestyle modifications–A new dimension in glaucoma management	no	study design / no quantitative data under the endpoints of interest
Amle	2022	380	Journal for Research in Applied Scie	Bioactive herbal medicine use for eye sight: a meta analysis	no	study design / no quantitative data under the endpoints of interest
Liu	2022	381	Journal of Ethnopharmacology	Leaves, seeds and exocarp of Ginkgo biloba L. (Ginkgoaceae): A Comprehensive Review of Traditional Uses, phytochemistry, pharmacology, resou	no	study design / no quantitative data under the endpoints of interest
Lauermann	2022	382	Clincal Ophthalmology	Risk factors for severe bleeding complications in glaucoma surgery and the role of antiplatelet or anticoagulant agents	no	study design / no quantitative data under the endpoints of interest
Feng	2023	383	Cells	The Role of Retinal Ganglion Cell Structure and Function in Glaucoma	no	not on topic
Boccaccini	2023	384	Molecular Aspects of Medicine	Novel frontiers in neuroprotective therapies in glaucoma: Molecular and clinical aspects	no	not on topic
Stuart	2023	385	Molecular Aspects of Medicine	Towards modifying the genetic predisposition for glaucoma: An overview of the contribution and interaction of genetic and environmental factors	no	not on topic
Butola	2023	386	Journal for Research in Applied Scie	Recent Approaches of Ocular Disease and Its Herbal Product Treatment: An Updates	no	not on topic
Gillmann	2023	387	The Science of Glaucoma Managem	Neuroprotection and neuroenhancement	no	not on topic
Luo	2023	388	Science of Advanced Materials	Effects of Ginkgo biloba Extract on Cognitive Function Recovery and Inflammatory Factors in Rats After Anesthesia	no	not on topic
Lakshmanan	2023	389	Neural Regeneration Research	Potential role of Lycium barbarum polysaccharides in glaucoma management: evidence from preclinical in vivo studies	no	not on topic
Chen	2023	390	Planta Medica	Comparative Inhibitory Effects of Natural Biflavones from Ginkgo against Human CYP1B1 in Recombinant Enzymes and MCF-7 Cells	no	not on topic
Gherghel	2023	391	Eye	Barriers to IOP-independent treatments in glaucoma clinical trials	no	not on topic
Tanuj	2023	392	The Science of Glaucoma Managem	Meditation for glaucoma management: cellular mechanisms and clinical impact	no	not on topic
Buonfiglio	2023	393	Antioxidants	Oxidative stress: a suitable therapeutic target for optic nerve diseases?	no	not on topic
Rauschkolb-Olk	2023	394	Dissertation	Vergleich der zirkadianen Wirksamkeit von Tafluprost-Augentropfen (Taflotan sine) mit Latanoprost-Augentropfen (Xalatan) in der Therapie des Offenwinkelglaukoms und der okulären Hypertension	no	other / not on topic
Szumny	2023	395	OphthaTherapy	Glaucoma-new possibilities, new research	no	other / not on topic
Lindner	2023	396	Pharmaceuticals	Therapeutic potential of cannabinoids in glaucoma	no	other / not on topic
Vitiello	2023	397	BioMed Research International	Herbal and Natural Treatments for the Management of the Glaucoma: An Update	no	study design / no quantitative data under the endpoints of interest
Marando	2023	398	Seminars in Ophthalmology	Evidence for complementary and alternative therapies to treat glaucoma	no	study design / no quantitative data under the endpoints of interest
Hou	2023	399	Scientific reports	Ginkgo biloba extracts improve choroidal circulation leading to suppression of myopia in mice	no	study design / no quantitative data under the endpoints of interest
Allison	2023	400	intechopen	Pharmacologic and Natural Therapeutics in Glaucoma Management	no	study design / no quantitative data under the endpoints of interest
Wu	2023	401	BMJ open	Efficacy and safety of Ginkgo biloba dropping pills in the treatment of coronary heart disease with stable angina pectoris and depression: study prot	no	study design / no quantitative data under the endpoints of interest
Li	2023	402	Journal of Pharmacy	Ginkgo biloba extracts (GBE) protect human RPE cells from t-BHP-induced oxidative stress and necrosis by activating the Nrf2-mediated antioxidar	no	study design / no quantitative data under the endpoints of interest
Ekici	2023	403	Molecular Aspects of Medicine	Advances in understanding glaucoma pathogenesis: a multifaceted molecular approach for clinician scientists	no	study design / no quantitative data under the endpoints of interest
Öztürk	2023	404	Literatür Eczacılık Bilimleri Dergisi	Therapeutic Applications of Ginkgo biloba L. Tree: Systemic Review	no	study design / no quantitative data under the endpoints of interest
Lin	2023	405	Soar suny edu	Effects of Gingko biloba on Systemic and Retinal Blood Circulation	no	study design / no quantitative data under the endpoints of interest
Suryono	2023	406	Medicinus	Neuroprotection in the Treatment of Glaucoma	no	study design / no quantitative data under the endpoints of interest
Qi	2023	407	Journal of Ethnopharmacology	Review on potential effects of traditional Chinese medicine on glaucoma	no	study design / no quantitative data under the endpoints of interest
Ng	2023	408	Antioxidants	Green tea catechins as therapeutic antioxidants for glaucoma treatment	no	study design / no quantitative data under the endpoints of interest
Ji	2023	409	Heliyon	A bibliometric and thematic analysis of the trends in the research on ginkgo biloba extract from 1985 to 2022	no	study design / no quantitative data under the endpoints of interest
Buonfiglio	2023	410	Pharmaceuticals	Immunomodulatory and antioxidant drugs in glaucoma treatment	no	study design / no quantitative data under the endpoints of interest
Dincel	2023	411	Journal of Kocaeli Health and Techr	A Review of Medicinal Properties of Ginkgo biloba L.	no	study design / no quantitative data under the endpoints of interest
Nguyen	2023	412	StatPearls	Ginkgo biloba	no	study design / no quantitative data under the endpoints of interest
Salvetat	2023	413	Pharmaceuticals	Pharmaceutical approaches to Normal tension Glaucoma	no	study design / no quantitative data under the endpoints of interest
Srivastava	2023	414	UP Journal of Ophthalmology	Recent updates on medical management of Glaucoma.	no	study design / no quantitative data under the endpoints of interest
Shen	2023	415	Neural Regeneration Research	Regulatory mechanisms of retinal ganglion cell death in normal tension glaucoma and potential therapies	no	study design / no quantitative data under the endpoints of interest
Kumar	2024	416	Herbal Medicine Phytochemistry	Evidence from the Use of Herbal Medicines in the Management and Prevention of Common Eye Diseases	no	not on topic
